# Efficacy of a Novel Class of RNA Interference Therapeutic Agents

**DOI:** 10.1371/journal.pone.0042655

**Published:** 2012-08-15

**Authors:** Tomohiro Hamasaki, Hiroshi Suzuki, Hisao Shirohzu, Takahiro Matsumoto, Corina N. D'Alessandro-Gabazza, Paloma Gil-Bernabe, Daniel Boveda-Ruiz, Masahiro Naito, Tetsu Kobayashi, Masaaki Toda, Takayuki Mizutani, Osamu Taguchi, John Morser, Yutaka Eguchi, Masahiko Kuroda, Takahiro Ochiya, Hirotake Hayashi, Esteban C. Gabazza, Tadaaki Ohgi

**Affiliations:** 1 BONAC Corporation, BIO Factory 4F, Aikawa, Kurume, Fukuoka, Japan; 2 Department of Immunology, Mie University Graduate School of Medicine, Mie, Japan; 3 Department of Pulmonary and Critical Care Medicine, Mie University Graduate School of Medicine, Mie, Japan; 4 National Cancer Center Research Institute, Tokyo, Japan; 5 Department of Molecular Pathology, Tokyo Medical University, Tokyo, Japan; 6 Laboratory of Molecular Genetics, Department of Medical Genetics, Osaka University Graduate School of Medicine, Osaka, Japan; 7 Division of Hematology, Stanford University School of Medicine, Stanford, California, United States of America; University of California Irvine, United States of America

## Abstract

RNA interference (RNAi) is being widely used in functional gene research and is an important tool for drug discovery. However, canonical double-stranded short interfering RNAs are unstable and induce undesirable adverse effects, and thus there is no currently RNAi-based therapy in the clinic. We have developed a novel class of RNAi agents, and evaluated their effectiveness *in vitro* and in mouse models of acute lung injury (ALI) and pulmonary fibrosis. The novel class of RNAi agents (nkRNA®, PnkRNA™) were synthesized on solid phase as single-stranded RNAs that, following synthesis, self-anneal into a unique helical structure containing a central stem and two loops. They are resistant to degradation and suppress their target genes. nkRNA and PnkRNA directed against TGF-β1mRNA ameliorate outcomes and induce no off-target effects in three animal models of lung disease. The results of this study support the pathological relevance of TGF-β1 in lung diseases, and suggest the potential usefulness of these novel RNAi agents for therapeutic application.

## Introduction

RNA interference (RNAi) is a natural mechanism for silencing gene expression that has been recently the focus of considerable attention for its potential application for the development of new drugs. During the process of RNAi, intracellularly introduced double-stranded (ds) RNA is cleaved into small interfering (si) RNA duplexes (19∼21 base pairs) that are incorporated into a protein complex called the RNA-induced silencing complex (RISC), which unwinds the two siRNA strands, retaining one strand to allow the recognition and sequence-specific degradation of mRNA [1]. RNAi-based therapy may provide several advantages over conventional therapeutic approaches using small molecules and monoclonal antibodies [2]. Unlike traditional pharmaceutical drugs, RNAi therapeutic agents can inhibit all classes of gene targets with high selectivity and potency, can provide personalized therapy, can be easily synthesized and the steps of lead identification and optimization are rapid [3], [4]. The proof-of-concept regarding the therapeutic applicability of RNAi was given by several *in vivo* studies performed in animal models of human disease. Inhibition of the expression of targeted genes and therapeutic efficacy have been successfully achieved with either local or systemic administration of siRNA in experimental animal models with diseases in the eyes, nervous system, viral infection, kidneys, liver, lungs or intestines. At present, there are more than ten RNAi-based drugs in clinical development [3].

Since its discovery a decade ago, RNAi has been widely used for functional gene studies in biomedical research and, currently, many efforts are being dedicated to harnessing their desirable properties for the development of new therapeutic drugs [5]. However, previous investigations made clear the existence of several obstacles needed to be overcome before their routine application in the clinic. Although siRNAs are more stable than ssRNAs, they are promptly degraded by nucleases when they are administered systemically [1]. The half-life of naked siRNAs is very short because they are rapidly degraded by nucleases from the serum and/or extracellular fluids [6]. Chemical modifications at specific positions have been shown to improve stability but they may attenuate the suppressive activity of siRNAs [7]. The cost of large-scale production is another hurdle to the clinical application of chemically modified or unmodified siRNA agents [8]. Moreover, the systemic administration of naked or modified siRNAs may induce undesirable off-target effects by activating the innate immune system via TLR-dependent or independent mechanisms leading to increased secretion of inflammatory cytokines (IFN-α, IFN-β) [9]. Appearance of these undesirable events has been reported to be sequence- and cell-type-dependent [10], [11]; many RNAi-based drugs have been withdrawn from clinical trials at early phase because the appearance of flu-like symptoms typical of an immune response [12]. Therefore, to achieve the ultimate goal of RNAi therapy, safety, effectiveness and delivery systems will need to be improved.

Pulmonary fibrosis is a chronic intractable disease and currently there are more than 5 million patients worldwide [13]. Transforming growth factor (TGF)-β1 plays critical roles in the pathogenesis of the disease by stimulating the proliferation of fibroblasts, by stimulating the secretion of extracellular matrix components and the recruitment and differentiation of myofibroblasts [14]. Several studies have also shown that TGF-β1 is implicated in the pathogenesis of acute lung injury (ALI) or acute respiratory distress syndrome (ARDS), which is another devastating disease with an overall mortality rate of 30∼40% [15]. TGF-β1 can directly increase the permeability of both alveolar epithelial cells and pulmonary artery endothelial cells, and can decrease the function of ion channels.

In the current study, to overcome some of the drawbacks faced by current siRNA-based therapy, we developed a novel class of RNAi therapeutic agents. In addition, using this new technology, we prepared novel RNAi agents directed against TGF-β1 and their inhibitory activity was compared to canonical siRNA using *in vitro* assays and animal models of ALI and pulmonary fibrosis.

## Results

### Synthesis, structure and suppressive activity of novel classes of RNAi agents

**Figure 1:**
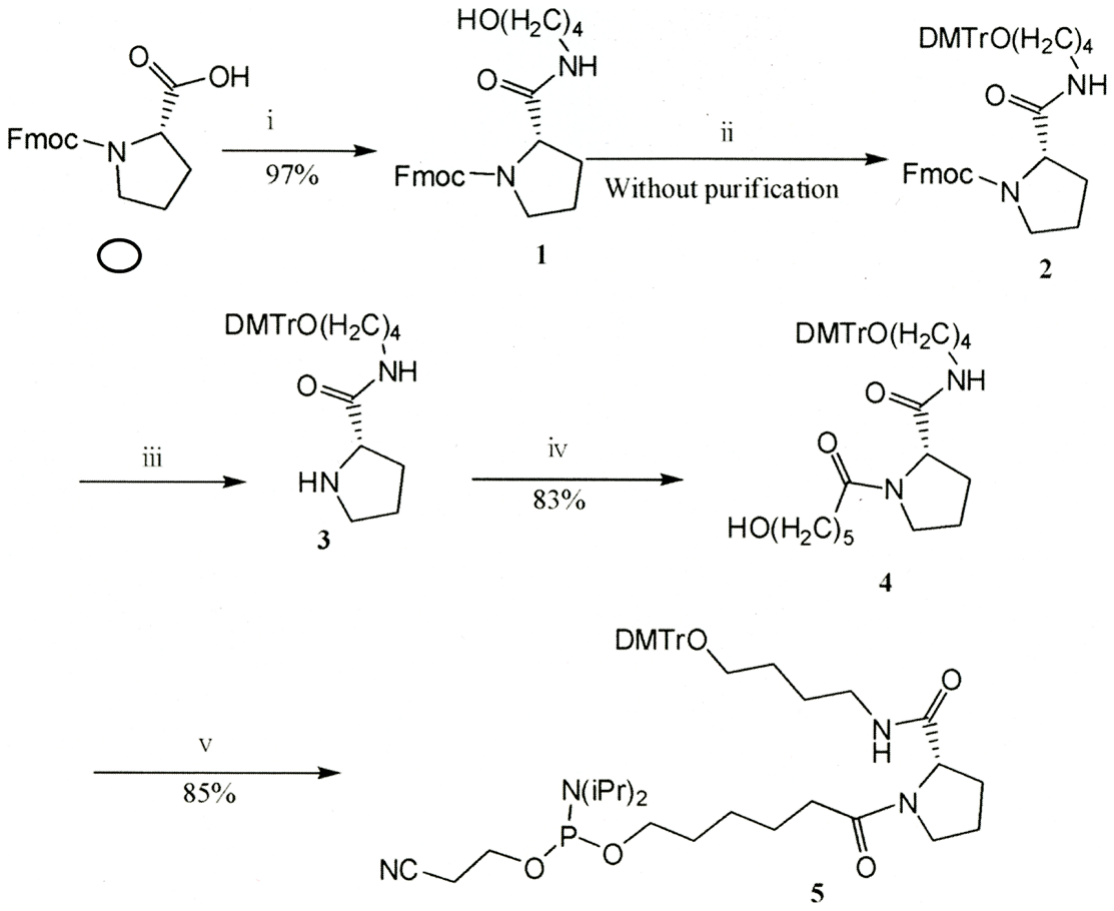
Synthesis of proline diamide amidite. Compounds 1, 2, 3 and 4 were sequentially prepared to synthesize proline diamide amidite (compound 5). (i) 4-Aminobutanol/N,N′-dicyclohexylcarbodiimide/1-hydroxybenzotriazole; (ii) 4,4′-dimethoxytrityl chloride/pyridine; (iii) piperidine/N,N-dimethylformamide; (iv) 6-Hydroxyhexanoic acid/1-ethyl-3-(3-dimethylaminopropyl) carbodiimide hydrochloride/1-hydroxybenzotriazole/triethylamine; (v) 2-cyanoethyl N,N,N′,N″0tetraisopropylphosphordiamidite/diisopropylammonium tetrazolide.

**Figure 2:**
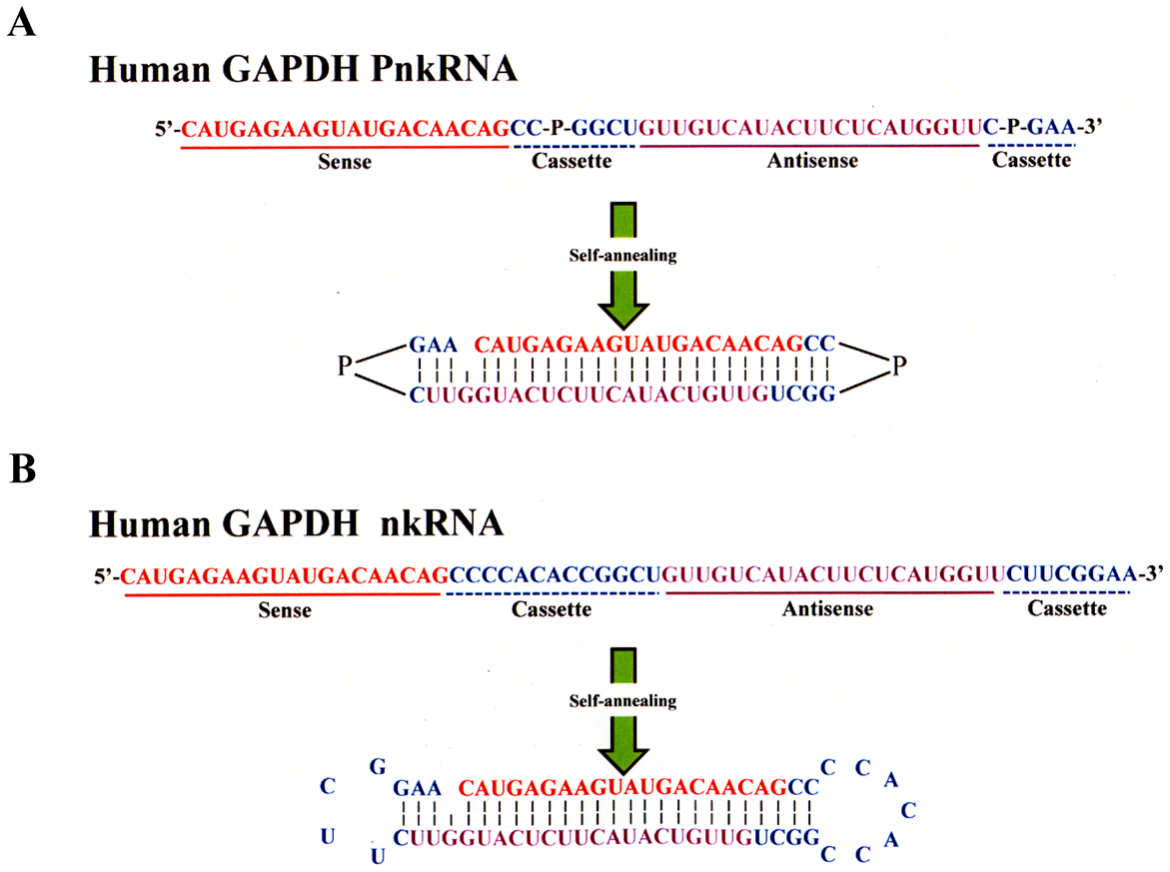
Structure of novel RNAi therapeutic agents. Both nkRNA (A) and PnkRNA (B) were prepared as single-stranded RNA oligomers that then self-anneal as shown. Nucleotides in red indicate sense strand of the target (GAPDH), nucleotides in violet are the antisense strand and nucleotides in blue are the loop cassettes; P indicates a proline derivative.

The RNAi agents were synthesized as single-stranded RNAs on solid phase as described under material and method section. Proline diamide amidites were prepared and used to incorporate two proline derivatives into one RNA oligomer (**Figure 1**). The human GAPDH was selected as a representative RNAi target. A long (51 mer) single-stranded human GAPDH RNA oligonucleotide (**Table 1**) that contains two proline derivatives (P) was synthesized. We discovered that this RNA oligomer with proline derivatives self-anneals into a structure that has a central stem formed by the sense and antisense nucleotides, an unpaired site and a nucleotide loop at the left (5′-CC-P-GGCU-3′) and right (5′C-P-GAA-3′) ends containing a proline derivative (**Figure 2A**). This RNA was named PnkRNA.

**Table 1:** Sequence of PnkRNA, nkRNA and siRNA directed against human GAPDH mRNA.

RNA class	Sequence	Mass	Purity (%)
Target siRNA	:5′- CCAUGAGAAGUAUGACAACAG -3′ (sense)/5′-GUUGUCAUACUUCUCAUGGUU-3′ (antisense)	n.d.	n.d.
Scrambled siRNA	:5′- GCCAUCAACGAUAAGUGAAAG -3′ (sense)/5′-UUCACUUAUCGUUGAUGGCUU-3′ (antisense)	n.d.	n.d.
Target PnkRNA dn1	:5′- CAUGAGAAGUAUGACAACAGCC-P-GGCUGUUGUCAUACUUCUCAUGGUUC-P-GAA-3′	17056.6	92.5
Scrambled PnkRNA	:5′- CCAUCAACGAUAAGUGAAAGCC-P-GGCUUUCACUUAUCGUUGAUGGCUUC-P-GAA-3′	17017.1	91.4
Target nkRNA dn1	:5′- CAUGAGAAGUAUGACAACAGCCCCACACCGGCUGUUGUCAUACUUCUCAUGGUUCUUCGGAA-3′	19779.9	96.9
Scrambled nkRNA	:5′-CCAUCAACGAUAAGUGAAAGCCCCACACCGGCUUUCACUUAUCGUUGAUGGCUUCUUCGGAA-3′	19739.3	92.2

A long (62 mer) single-stranded human GAPDH RNA oligonucleotide without the incorporation of proline derivatives was also synthesized. This single-stranded GAPDH RNA oligonucleotide was also found to self-anneal into a structure containing a central stem, a loop at the left and right ends and an unpaired site (**Figure 2B**). The central stem contains the sense and antisense nucleotides, the left loop is partially formed by a nucleotide cassette containing the sequence 5′-CUUCGGAA-3′, and the right loop is partially formed by a nucleotide cassette containing 5′-CCCCACACCGGCU-3′. RNAs with this structure were named nkRNAs.

**Figure 3:**
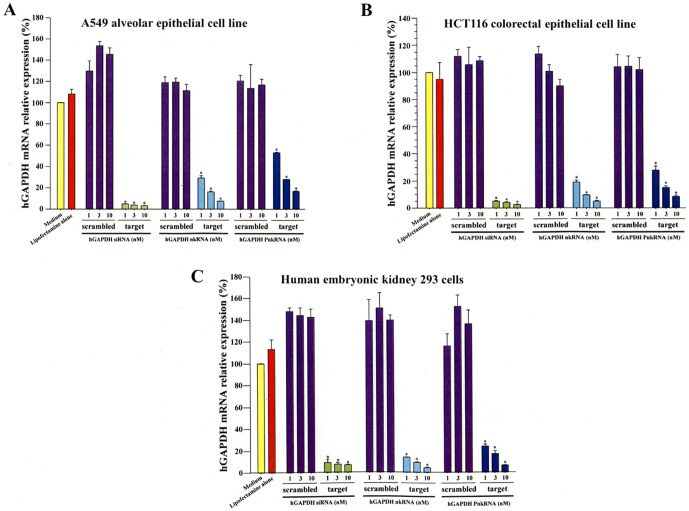
Inhibition of human GAPDH expression by novel RNAi agents. siRNA, nkRNA and PnkRNA directed against human (h)GAPDH significantly inhibited GAPDH gene expression in a dose-dependent fashion in A549 (A), HCT116 (B) and HEK 293 (C) cell lines. Statistical analysis by ANOVA. Data are expressed as the mean ± s.e.m. *p<0.0001 vs. control groups.

Representative HPLC chromatogram and mass spectrum of the novel RNAi agents against human GAPDH, human and mouse TGF-β1 is described in **Figure S1**. The inhibitory effect of nkRNA and PnkRNA directed against human GAPDH was evaluated and compared with canonical human GAPDH siRNA. Similar to canonical siRNA, both nkRNA and PnkRNA showed strong suppressive activity of GAPDH expression in A549, HCT116 and HEK 293 cell lines (**Figure 3A, B, C**).

### Inhibitory activity depends on position of unpaired sites

**Figure 4:**
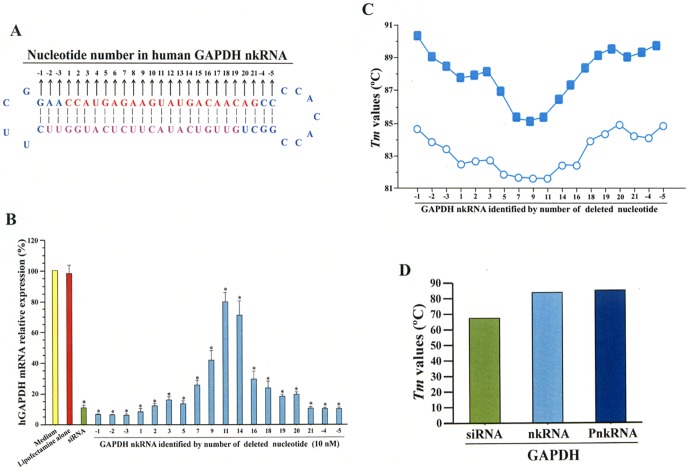
Position of deleted nucleotides affects the inhibitory activity of nkRNA. Numbering of nkRNA is shown in (A). Inhibition of human GAPDH mRNA expression in HCT 116 cells by GAPDH nkRNAs (10 nM) with deleted nucleotides at different positions; nkRNAs with deleted nucleotides near the right or left loop showed increased inhibitory activity (B). The *Tm* values of the GAPDH nkRNAs with deleted nucleotides at different positions were measured (C); comparison between (B) and (C) shows that the inhibitory activity of GAPDH nkRNAs is proportionally correlated with their *Tm* values. Comparative *Tm* values of siRNA, nkRNA and PnkRNA (D). *p<0.05 vs. control groups. Closed squares indicate samples diluted in phosphate-buffered saline; open circles indicate samples diluted in phosphate buffer.

Both nkRNA and PnkRNA have a nicked site that is unpaired on their sense strands. We investigated if the location of that unpaired site is important by preparing self-annealed nucleic acids with unpaired sites at different positions on the sense strand of nkRNA directed against GAPDH mRNA (**Table S1;** **Figure 4A**). Their inhibitory activity was evaluated in the HCT 116 cells. Deletion of nucleotides from the left (positions −1, −2, −3) and right (positions −4, −5) cassettes of nkRNA and from the sense strand near the left (positions 1, 2, 3, 5) and right (positions 19, 20, 21) loops of nkRNA was associated with strong inhibitory activity (**Figure 4B**).

The *Tm* value of nkRNA was significantly correlated with its respective inhibitory activity, suggesting that the suppressive effect of nkRNA depends on the deleted position (**Figure 4C**). The *Tm* values for nkRNA and PnkRNA directed against human GAPDH were higher than the *Tm* for siRNA directed against human GAPDH (**Figure 4D**).

### Weak suppressive activity of circular dumbbell-shaped RNA

**Figure 5:**
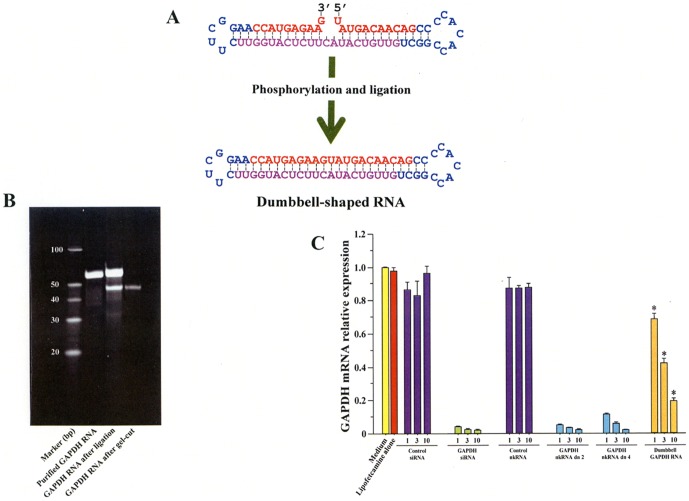
Preparation and *in vitro* activity of dumbbell-shape RNA with endless nucleotide. RNA was prepared as described under method in supplementary materials and the RNA 5′ end was phosphorylated by treating with T4 polynucleotide and the RNA 3′ and 5′ ends were ligated and converted to a circular type by treating with T4 RNA ligase (A). The product was confirmed by gel electrophoresis (B). HCT 116 cell lines were treated with varying concentrations of each RNA in the presence of lipofectamine and after 48 h, mRNA was extracted and the amount of hGAPDH was quantified by real time PCR (C). Data are expressed as the mean ± s.e.m. *p<0.0001 vs. respective concentration of siRNA, nkRNA dn 2 and nkRNA dn 4.

A circular dumbbell-shaped RNA was previously reported to have stronger suppressive activity than canonical siRNA [16]. Dumbbell type nkRNA directed against human GAPDH was prepared to compare its inhibitory activity with the corresponding siRNA and nkRNA against human GAPDH. Treatment of RNA 5′ and 3′ ends with T4 polynucleotide kinase and T4 RNA ligase led to formation of a 50 base band on the PAGE that corresponds to the dumbbell-shaped RNA of GAPDH (**Figure 5A, B**). When the ligated band was eluted and tested for reduction of GAPDH mRNA in human HCT 116 cells, siRNA and nkRNA had a 10-fold stronger suppressive activity than the equivalent dumbbell RNA (**Figure 5C**).

### *In vitro* activity of novel RNAi agents against TGF-β1

In order to test the novel RNAi agents against a gene involved in disease, nkRNA and PnkRNA directed against mouse TGF-β1 with deleted nucleotides at different positions were prepared (**Table S2**) and their suppressive effect was compared. TGF-β1 nkRNA and PnkRNA with deleted nucleotides at positions −2 and −3 showed stronger inhibitory activity than RNAi agents with deletion at positions 1 or 1+2 in Hepa 1–6 liver cell lines (**Figure S2A**). nkRNA and PnkRNA against mouse TGF-β1 also showed strong inhibitory activity in LA-4 lung epithelial cells (**Figure S2B**).

### Digestion by Dicer

Cleavage of the two nucleotide overhangs at the 3′ end of a long double-stranded RNA by Dicer, a type III ribonuclease, leads to the intracellular release of functional 21 to 23 mer siRNAs [1]. To demonstrate that nkRNA and PnkRNA can be digested by Dicer, nkRNA dn1 and PnkRNA dn1 directed against mouse TGF-β1 (**Table S2**) were incubated with Dicer. Digestion of nkRNA and PnkRNA by Dicer led to the formation of 21∼22 mer dsRNA (**Figure S3A, B, C** and **Table S3**), suggesting that the digestion products of these novel RNAi agents resemble siRNA and explain their significant suppressive activity.

### *In vitro* stability of RNAi agents

**Figure 6:**
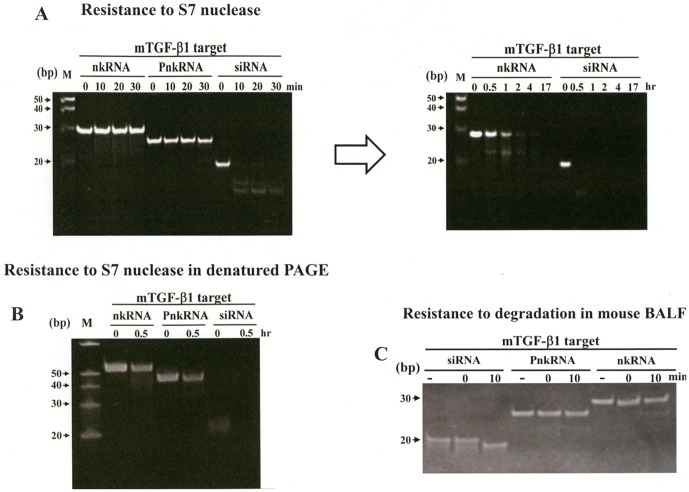
Stability of nkRNA, PnkRNA and siRNA directed to mouse TGF-β1. For evaluating *in vitro* stability, each RNA was incubated at 37°C in the presence of S7 nuclease (A, B) and then separated on polyacrylamide gel electrophoresis (PAGE). Both nkRNA and PnkRNA were more resistant to degradation than siRNA. Stability of the therapeutic agents in bronchoalveolar lavage fluid (BALF) from mice was also evaluated; each RNA was added to 50 µl of BALF, incubated at 37°C and then separated on PAGE (C). siRNA was completely degraded but nkRNA and PnkRNA remained intact after 10 min (C). -; indicates lane loaded with RNA that was not added to BALF.

The stability of the RNAi agents, siRNA, nkRNA and PnkRNA against mouse TGF-β1 (**Table S4**) to degradation by nucleases was compared. Each RNA was incubated in the presence of S7 nuclease and the degree of degradation was followed overtime. While siRNA was completely degraded after 10 min of incubation with S7 nuclease, nkRNA and PnkRNA were still intact after 30 min; incubation for longer time showed that nkRNA was still partially intact even after 1 h of incubation with S7 nuclease (**Figure 6A**). Analysis of the RNAs after S7 degradation on denatured PAGE disclosed similar results (**Figure 6B**).

### Stability of RNAi agents in lung fluid

To evaluate the stability of the therapeutic agents close to *in vivo* conditions, siRNA, nkRNA or PnkRNA directed against mouse TGF-β1 was incubated in BALF taken from mice and the grade of degradation was assessed by PAGE. siRNA was completely degraded but both nkRNA and PnkRNA remained intact after 10 min of incubation in BALF (**Figure 6C**).

### Efficacy of nkRNA in wild type mouse with ALI

**Figure 7:**
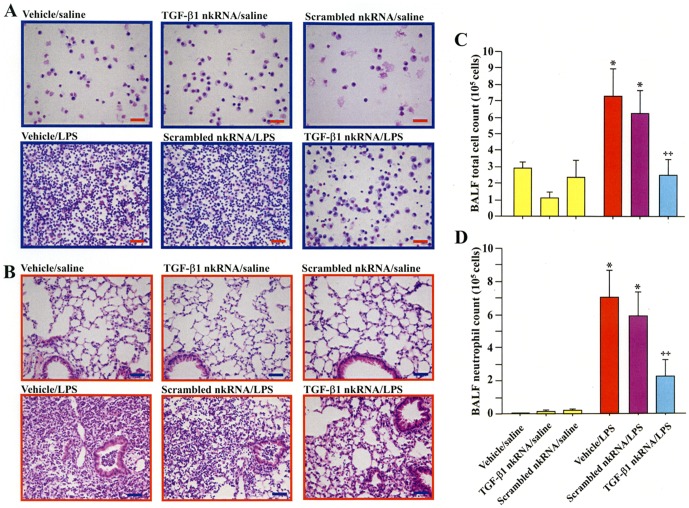
TGF-β1 nkRNA reduces inflammation in acute lung injury. Inflammatory cells in bronchoalveolar lavage fluid (BALF) (A) and lung tissue (B) were reduced in LPS-instilled mice treated with TGF-β1 nkRNA compared to those treated with vehicle or control RNA. The total cell count (C) and the number of neutrophils (D) in BALF were significantly reduced in LPS-instilled mice treated with TGF-β1 nkRNA compared to those treated with vehicle or control RNA. The scale bars indicate 50 µm. Statistical analysis by ANOVA. Data are expressed as the mean ± s.e.m. *p<0.05 vs controls. ‡p<0.05 vs untreated group.

To investigate if these novel siRNAs possess *in vivo* activity, mice were pre-treated with mouse TGF-β1 nkRNA dn 1 by i.t. instillation before LPS or saline administration and sacrificed after 24 h. The inflammatory cells in BALF (**Figure 7A**) and lung tissue (**Figure 7B**) were conspicuous in mice that received i.t. instillation of LPS and vehicle or scrambled nkRNA compared to control groups. Mice pre-treated with TGF-β1 nkRNA showed decreased inflammatory cells in BALF and lung tissue compared to vehicle/LPS and scrambled nkRNA/LPS groups (**Figure 7A, B**). Cell counting showed that the total cell count and the number of neutrophils in BALF were significantly increased in mice treated with LPS and vehicle (vehicle/LPS) or scrambled nkRNA (scrambled nkRNA/LPS) compared to control mice (**Figure 7C, D**). Mice treated with TGF-β1 nkRNA have significantly decreased total cell and neutrophil counts compared to both vehicle/LPS and scrambled nkRNA/LPS group; no significant changes in BALF cells were observed in mice from the saline/TGF-β1 nkRNA group compared to controls (**Figure 7C, D**).

**Figure 8:**
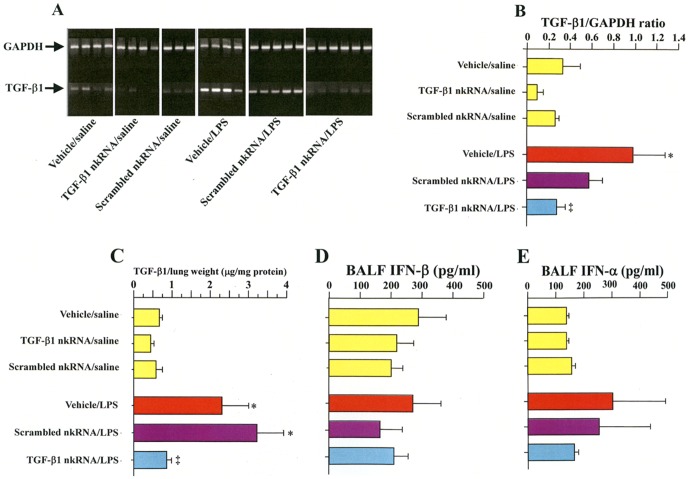
TGF-β1 nkRNA decreases target expression but had no effect on interferon (IFN) expression in acute lung injury. The mRNA (A, B) and protein (C) expression of TGF-β1 were significantly decreased in LPS-instilled mice treated with TGF-β1 nkRNA compared to those treated with vehicle or control RNA. The concentration of IFN-α (D) and IFN-β (E) in BALF was not affected by the treatment. Statistical analysis by ANOVA. Data are expressed as the mean ± s.e.m. *p<0.05 vs controls. ‡p<0.05 vs untreated group.

The mRNA expression (**Figure 8A, B**) and protein level (**Figure 8C**) of TGF-β1 in the lungs were increased in mice receiving LPS and vehicle or scrambled nkRNA compared to control mice but they were significantly suppressed in mice treated with TGF-β1 nkRNA. In addition, the concentration of IFN-α and IFN-β in BALF was not significantly changed among all mouse groups (**Figure 8D, E**).

### Comparative efficacy of RNAi agents in ALI in reducing TGF-β1 mRNA and protein levels

The inhibitory activity of the RNAi agents in the ALI model was compared using a dose of 100 µg per mouse. The TGF-β1 expression tended to be much more strongly suppressed by PnkRNA and nkRNA compared to siRNA (**Figure S4A**). To confirm that TGF-β1 mRNA was degraded, 5′-RACE assay was performed and the results confirmed degradation of TGF-β1 mRNA by each RNAi agent (**Figure S4B**).

### Efficacy of RNAi agents in human TGF-β1 TG mice with ALI

**Figure 9:**
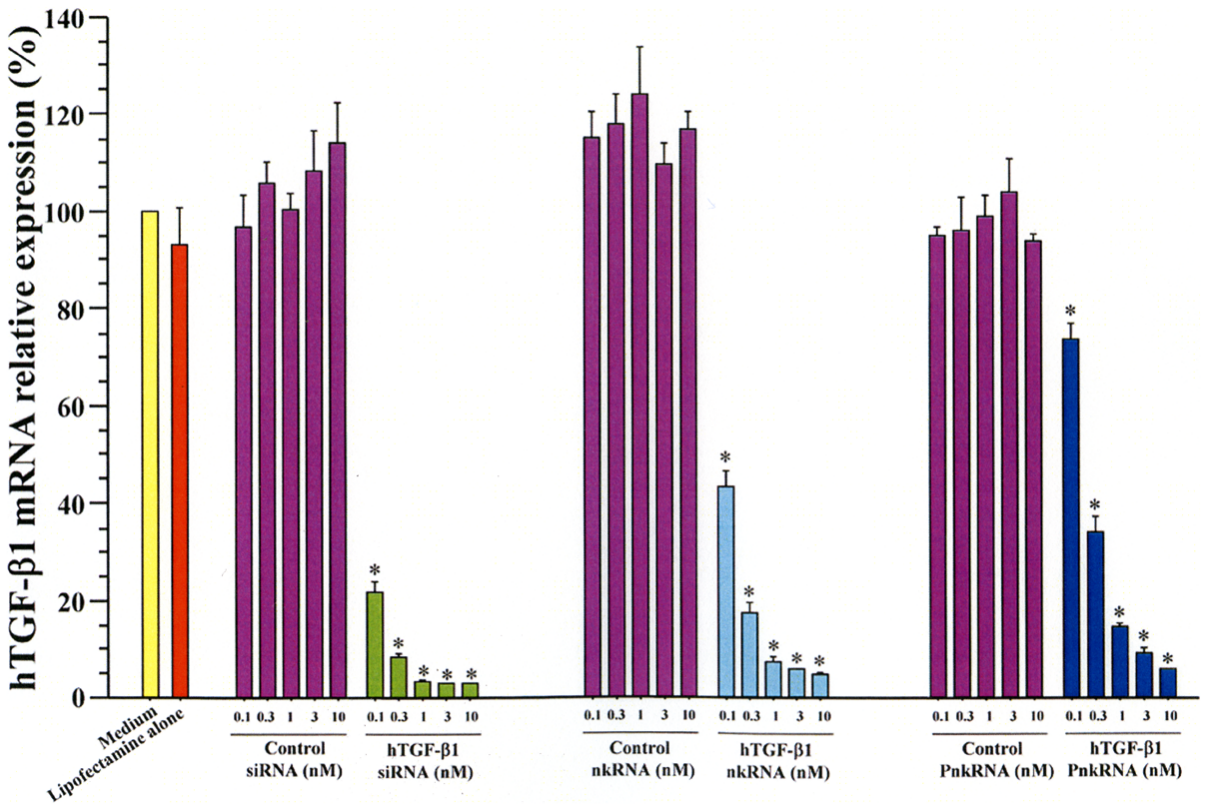
Inhibitory activity of siRNA, nkRNA dn -2 and PnkRNA dn -2 directed against human TGF-β1. The in vitro inhibitory activity of siRNA, nkRNA dn -2 and PnkRNA dn -2 against TGF-β1 was significant compared to controls in A549 cells. Statistical analysis by ANOVA. Data are expressed as the mean ± s.e.m. *p<0.05 vs controls.

**Figure 10:**
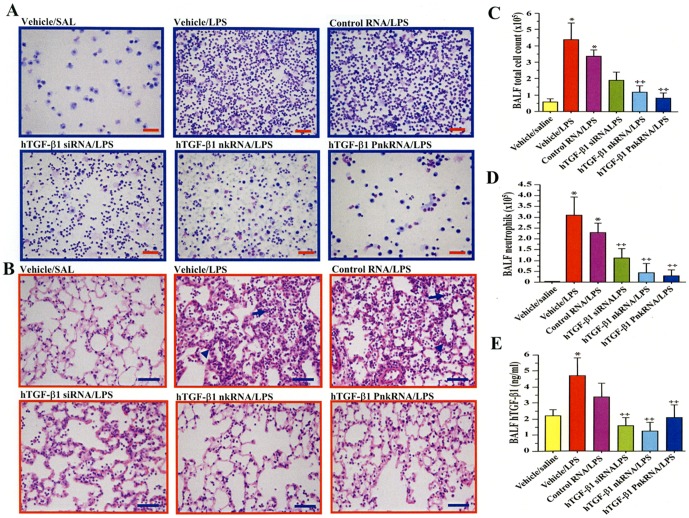
hTGF-β1 nkRNA and PnkRNA inhibit acute lung injury in human TGF-β1 TG mice. Inflammatory cells were conspicuous in BALF from vehicle/LPS and control RNA/LPS groups compared to vehicle/SAL group and mice treated with each RNAi therapeutic agent (A). Histological findings of the lung showed increased infiltration of inflammatory cells (arrows), alveolar thickening (arrow heads) and edema in vehicle/LPS and control RNA/LPS groups compared to the vehicle/SAL group and mice treated with each RNAi agent (B). The BALF total cell count was significantly decreased by nkRNA and PnkRNA and the number of neutrophils by siRNA, nkRNA and PnkRNA compared to vehicle/LPS mice (C, D). The BALF concentration of human TGF-β1 was significantly reduced by each nucleic acid agent compared to untreated mice (E). The scale bars indicate 50 µm. Statistical analysis by ANOVA. Data are expressed as the mean ± s.e.m. *p<0.05 vs controls. ‡p<0.05 vs untreated group.

To evaluate therapeutic effect in hTGF-β1 TG mice with ALI, siRNA, nkRNA dn −2 and PnkRNA dn −2 directed against human TGF-β1 were prepared (**Table S5**). All of the therapeutic agents, siRNA, nkRNA dn −2 and PnkRNA dn −2, possessed the predicted suppressive effect against human TGF-β1 as confirmed by an *in vitro* assay using human A549 cells (**Figure 9**). Human TGF-β1 mice were pre-treated with one of siRNA, nkRNA or PnkRNA and then received i.t. instillation of LPS. Microscopic observation of BALF cells showed increased inflammatory cells in the vehicle/LPS and control RNA/LPS groups compared to the vehicle/SAL group and mice treated with siRNA, nkRNA or PnkRNA against hTGF-β1 (**Figure 10A**). Acute inflammatory changes in the lung tissues characterized by alveolar thickening, edema and infiltration of inflammatory cells were conspicuous in untreated mice compared to mice treated with any of the RNAi agents (**Figure 10B**). The total cell count and the number of neutrophils in BALF were significantly increased in mice receiving instillation of vehicle (vehicle/LPS) or control RNA (control RNA/LPS) and LPS compared to control mice (vehicle/SAL), but they were significantly decreased in the group of mice treated with siRNA (TGF-β1 siRNA/LPS), nkRNA (TGF-β1 nkRNA/LPS) or PnkRNA (TGF-β1 PnkRNA/LPS) directed against human TGF-β1 compared to untreated mice (**Figure 10C, D**). The BALF concentration of hTGF-β1 was also increased in the vehicle/LPS group but it was significantly reduced by treatment with siRNA, nkRNA or PnkRNA directed against TGF-β1 (**Figure 10E**).

### RNAi agents ameliorate pulmonary fibrosis

**Figure 11:**
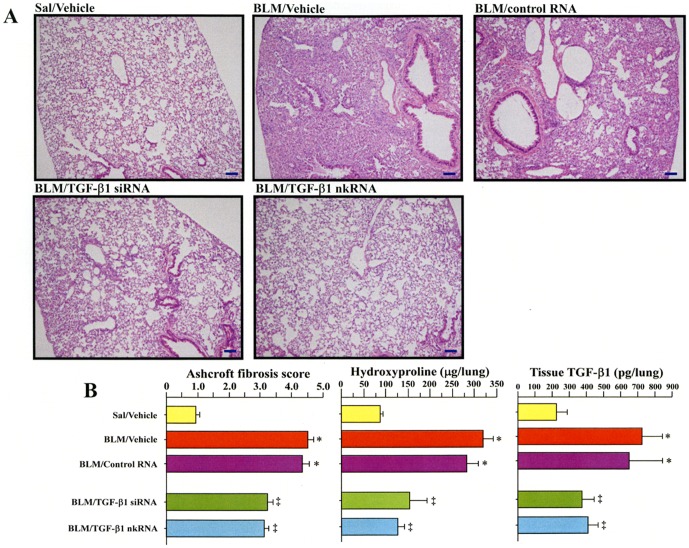
Mouse TGF-β1 nkRNA improves lung fibrosis. Mice with lung fibrosis induced by bleomycin were treated with mTGF-β1 siRNA, mTGF-β1 nkRNA and control RNA. Mice infused with s.c. saline served as control. TGF-β1 siRNA and nkRNA significantly inhibited pulmonary fibrosis as shown by the lung pathological findings (A) Ashcroft score and hydroxyproline content (B). The concentration of TGF-β1 was significantly inhibited by both TGF-β1 siRNA and nkRNA treatment (B). The scale bars indicate 100 µm. Statistical analysis by ANOVA. Data are expressed as the mean ± s.e.m. *p<0.05 vs. control groups. ‡p<0.05 vs untreated group.

Lung fibrosis was induced by BLM administered through osmotic mini pumps placed subcutaneously in the back of wild type animals. Mice were treated by instillation of 100 µg per mouse of each siRNA and nkRNA dn −2 against mouse TGF-β1. Both siRNA and nkRNA significantly suppressed collagen deposition in the lungs compared to untreated mice as demonstrated by the significant decrease in fibrosis score and hydroxyproline content of the lungs in BLM/TGF-β1 siRNA and BLM/TGF-β1 nkRNA groups compared to BLM/vehicle and BLM/control RNA groups (**Figure 11A, B**). Treatment with either RNAi agents was also associated with significant decreases in concentration of TGF-β1 in the lungs compared to untreated mice (**Figure 11B**).

## Discussion

In this study, we reported on the development of a novel class of RNAi therapeutic agents (nkRNA® & PnkRNA™). They are prepared as single stranded RNA that self-anneals into a unique structure containing a doubled stranded RNA with an unpaired site, bound at the right and left ends by an oligonucleotide loop or by a non-nucleotide molecule (proline derivative). These novel RNAi therapeutic agents require simple methods for *in vitro* synthesis, show significant effectiveness in disease models and are more stable than canonical siRNAs.

### Current challenges of RNAi-based therapy

Despite the attractive and promising aspects of RNAi for new drug discovery some hurdles still remain to be overcome before routine application in the clinic [5]. These obstacles include the lack of an appropriate system for efficient and safe delivery of the drug to the target tissue, the induction of undesirable off-target effects and immune-mediated toxicities [5]. In the current study, we developed a new class of RNAi agents showing superior resistance against nuclease degradation compared to canonical siRNA. Both nkRNA and PnkRNA remained intact even after hours of incubation with nucleases, whereas siRNA were already completely degraded just a few minutes after incubation in the same conditions; further, siRNA was completely degraded but nkRNA and PnkRNA remained intact after incubation in BALF from mice. The production of the novel RNAi agents is simple; because nkRNA and PnkRNA are synthesized as ssRNAs that spontaneously self-anneal they do not require an annealing step, so large-scale production at low cost is possible. Further, intratracheal instillation of the nkRNA and PnkRNA was not associated with off-target expression of inflammatory cytokines either in the mouse model of LPS-induced acute lung injury, suggesting that they might provide a solution to the safety concerns about off-target effects of canonical siRNAs.

### *In vitro* suppressive effect of novel RNAi agents

To find a solution to the stability problem, Abe et al developed a circular RNA containing a central double-stranded stem closed by a nucleotide loop on each side of the structure, the so called dumbbell RNAs [17]. To prepare the endless helical RNAs, the authors first synthesized dsRNA and then closed it at both ends with helical sequences using T4 RNA ligases [16] and found that the dumbbell-shaped RNA was more stable and more efficient *in vitro* than canonical siRNAs [16]. Unlike the dumbbell-shaped RNAs, we reported here nkRNA and PnkRNA that can be synthesized as ssRNAs that self-anneal into a helical structure but one that contains an unpaired site (3′ and 5′ ends) on the sense strands. To clarify whether endless helical RNAs cause increased inhibition, we prepared dumbbell-type RNAs and compared their suppressive ability with nkRNAs *in vitro*. We found that dumbbell-type RNA has lower inhibitory activity than nkRNA which may be explained by the degree of incorporation into RISC and degradation by Dicer.

When human GAPDH nkRNAs with deleted nucleotides at different positions in the sense strands were compared in their inhibitory activities, nkRNAs with deleted nucleotides near the loops showed more suppression than nkRNAs lacking nucleotides near the middle site of the central stem. This phenomenon was not gene-dependent because similar results were obtained when the inhibitory effect of several mouse TGF-β1 nkRNAs with different nucleotide deletions was evaluated. Interestingly, the *Tm* values of the nkRNAs were significantly correlated with their *in vitro* inhibitory activity on gene expression, and the *Tm* values of nkRNA and PnkRNA were increased compared to those of canonical siRNAs. Overall, this suggests that stability at high *Tm* is the determing factor for the differences in suppressive activity observed between nkRNAs with nucleotides deleted at different positions, and between nkRNA, PnkRNA and canonical siRNAs.

### Inhibitory effects of nkRNA and PnkRNA in ALI

To evaluate the potential utility of the new RNAi agents for clinical application, we prepared nkRNA and PnkRNA directed against mouse or human TGF-β1 and used them to treat wild type or human TGF-β1 TG mice with LPS-induced ALI. Several evidence has shown the implication of TGF-β1 in the pathogenesis of ALI/ARDS [18], [19], [20], [21], [22]. In the present study, we found that i.t. instillation of nkRNA and PnkRNA directed against TGF-β1, decreased the RNA and protein expression levels of TGF-β1 while significantly inhibiting the infiltration of inflammatory cells in the mouse model of ALI. Interestingly, neither the expression of IFN-α nor IFN-β was increased by treatment with the RNAi agents. To corroborate that TGF-β1 inhibition RNAi therapy can improve disease under conditions similar to human disease, ALI was induced in hTGF-β1 TG mice and the effect of the novel RNAi agents directed against human TGF-β1 was assessed. The results showed similar beneficial effects in hTGF-β1 TG mice with ALI to those observed in their wild type counterparts, suggesting that RNAi agents would also inhibit the progression of the disease in humans.

### Inhibitory effect of nkRNA in pulmonary fibrosis

Several growth factors including platelet-derived growth factor, connective tissue growth factor and TGF-β1 play critical roles in the pathogenesis of the pulmonary fibrosis [14]. In a previous study, we showed that siRNA directed against growth factors significantly inhibit collagen deposition in the lung but, in particular, TGF-β1 siRNA was the strongest inhibitor of lung fibrosis [23]. In the present study, we evaluated the suppressive activity of nkRNA and PnkRNA directed against TGF-β1 on pulmonary fibrosis induced by BLM in wild type mice. The results showed a significant inhibition of the gene target leading to a reduction in collagen deposition in treated mice compared to controls, suggesting the effectiveness of these novel RNAi therapeutic agents against lung fibrosis.

### Conclusion

This report describes the *in vitro* and *in vivo* properties of a novel class of RNAi therapeutic agents (nkRNA® & PnkRNA™) that are more resistant to nuclease degradation. These novel RNAi agents directed against TGF-β1mRNA ameliorate outcomes and showed no off-target effects in wild type and human TGF-β1 TG mice with ALI and in wild type mice with pulmonary fibrosis, thereby supporting the pathological relevance of TGF-β1 in lung diseases. Thus these novel RNAi therapeutic agents are safe and may be tested for applications in the clinic.

## Materials and Methods

### Ethics Statement

The experimental protocol of this study was approved by the Mie University's Committee on Animal Investigation.

### Reagents

Dulbecco's modified Eagle medium (DMEM), RPMI-1640 and LPS from *Esherichia coli*, were purchased from Sigma (St Louis, MO). L-glutamine, vitamin solution, sodium pyruvate, penicillin/streptomycin and nonessential amino acids were purchased from Invitrogen (Carlsbad, Calif). Fetal bovine serum (FBS) was purchased from BioWhittaker (Walkersville, MD).

### Preparation of proline diamide amidite

Proline diamide amidite was synthesized and used to incorporate a proline derivative molecule into the long RNA oligomer. Synthesis of proline diamide amidite was performed following the scheme described in Figure 1. S-2-(4-hydroxybutylaminocarbonyl)-1-(9-fluorenylmethoxycarbonyl) pyrrolidine was prepared by dehydrating condensation of Fmoc-L-proline and 4-amino-1-butanol using dicyclohexylcarbodiimide and N-hydroxybenzotriazole (yield 97%), and then treated with dimethoxytrityl chloride in pyridine at room temperature forming S-2-(4-(4,4′-dimethoxytrityloxy)-butylaminocarbonyl)-1-(9-fluorenylmethoxycarbonyl)pyrrolidine. In this compound, deprotection of Fmoc was performed using piperidine in N, N-dimethylformamide at room temperature leading to formation of a pale yellow syrup (yield 98%) obtained after applying to silica gel column chromatography using a mixture of dichloromethane and methanol (9∶1+0.05% pyridine) as eluent. The dehydrating condensation of S-2-(4-(4,4′-dimethoxytrityloxy)-butylaminocarbonyl)pyrrolidine and 6-hydroxyhexanoic acid with 1-ethyl-3-(3-dimethylaminopropyl) carbodiimide hydrochloride and 1-hydroxybenzotriazole in dichloromethane at room temperature led to the formation of a pale yellow syrup (yield 83%) after applying to a silica gel column chromatography using a mixture of dichloromethane and methanol (9∶1+0.05% pyridine) as eluent. The mixture of S-2-(4-(4,4′-dimethoxytrityloxy)-butylaminocarbonyl)-1-(5-hydroxypentylcarbonyl) pyrrolidine, 2 – cyanoethyl - N, N, N′, N′-teraisopropylphosphordiamidite, and diisopropylammonium tetrazolide in acetonitrile was stirred for 4 h at room temperature; the reaction mixture was diluted with dichloromethane and then washed with saturated aqueous sodium bicarbonate and brine. After concentration of the organic layer in vacuum, the mixture was subjected to column chromatography using aminosilica with a mixture of n-hexane and acetone (7∶3+0.05%pyridine) as eluent. S-2-(4-(4,4′-dimethoxytrityloxy)-butylaminocarbonyl)-1-(5-(O-(2-cyanoethoxy)(diisopropylamino)phosphinoxypentylcarbonyl) pyrrolidine (proline diamide amidite) was obtained with 85% yield (HPLC purity 93%).

### Preparation of RNAi agents

High performance liquid chromatography (HPLC) analysis was performed using the Shimadzu LC-10A system (Shimadzu Corporation, Kyoto, Japan). XBridge OST C18 (1.7 µm, 4.6×50 mm) (Waters) column was used for reversed-phase HPLC analysis of short RNA oligomers and DNAPac PA-100 (4×250 mm) (DIONEX) column for anion-exchange HPLC analysis of long oligomers. RNA oligonucleotides were synthesized using commercially available controlled-pore glass solid supports placed in columns that were installed in the nucleic acid synthesizer Applied Biosystems Expedite model 8909 for the synthesis of nkRNA and PnkRNA and the nucleic acid synthesizer Applied Biosystems ABI 3900 for the synthesis of siRNA. The short RNA oligomers of siRNA (20∼21 mer) and the long RNA oligomers of nkRNA (62 mer) and PnkRNA (51 mer) were synthesized using standard phosphoramidites. Incorporation of a proline derivative molecule into the PnkRNA oligomer was performed using proline diamide amidite synthesized as described above (**Figure 1**).

Trichloroacetic acid diluted in dichloromethane was used as a detritylation solution and both amidite in acetonitrile and 5-benzylmercaptotetrazole as activating reagent were used in the coupling reaction. All commercially available reagents and solvents were used without further purification. Once synthesis was completed, the RNA oligonucleotides were purified by removing all protecting groups. The purity of the crude RNA product was confirmed by reverse-phase and anion-exchange HPLC and polyacrylamide gel electrophoresis. Mass was measured by using a liquid chromatography (ACQUITY UPLC) coupled with electrospray ionization-quadrupole-time-of-flight tandem mass spectrometer (LC-ESI-Q-Tof/MS) (SYNAPT G2 MS, Waters Corporation, Milford Massachusetts).

After ethanol precipitation, purified RNA oligomers for siRNAs were dissolved in HEPES buffer (30 mM HEPES-KOH, 100 mM potassium acetate, 2 mM magnesium acetate, pH 7.4) and purified nkRNA and PnkRNA oligomers were dissolved in RNase-free distilled water. Preparation of siRNAs was done by annealing the purified short complementary strands at 90°C for 10 min and then incubated at room temperature for 1 h. The long RNA oligomers of nkRNA and PnkRNA self-anneal and thus require no heating step. Duplex formation of the oligomers was confirmed by polyacrylamide gel electrophoresis.

### Cell culture and transfections

The human HCT 116 cells (DS Pharma Biomedical Co., Ltd., Japan) were cultured in McCoy's 5A medium (Invitrogen, Grand Island, NY) with 10% fetal bovine serum (FBS), 50 units/mL penicillin, and 50 µg/mL streptomycin. The human A549 cells and HEK 293 cells were obtained from the American Type Culture Collection (Rockville, MD) and cultured in DMEM medium containing 10% heat-inactivated FBS, 50 µg/mL penicillin, 50 µg/mL streptomycin, 2 mM L-glutamine and 0.1 mM nonessential amino acids. The mouse hepatocellular carcinoma Hepa1–6 cells (RIKEN BioResource Center, Japan) were cultured in DMEM (Invitrogen) with 10% heat-inactivated FBS, 50 units/mL penicillin, and 50 µg/mL streptomycin. The mouse lung adenoma cell line LA-4 from Dainippon Sumitomo Pharma (Tokyo, Japan) was cultured in F-12 Ham's medium (Sigma-Aldrich, St. Louis, MO). Cells were incubated at 37°C in a humidified 5% CO_2_ atmosphere. Confluent cells were harvested by brief exposure to 0.025% trypsin-0.02% EDTA in Hepes-buffered saline (50 mM Hepes, 150 mM NaCl at pH 7.4) and passaged after 5 to 7 days.

Human HCT 116, A549 or HEK293 cells were seeded on 24-well plates (1×10^5^ cells/well), and incubated at 37°C, in humidified 5% CO_2_ for 24 hours. The culture medium was replaced by 400 µL of McCoy's 5A medium containing 10%FBS without antibiotics, and then transfected with 98.5 µL siRNA or the novel RNAi agents (nkRNA®, PnkRNA™) directed against human glyceraldehyde 3-phosphate dehydrogenase (GAPDH) in Opti-MEM (Invitrogen) (final concentration: 1, 3, 10 nM), using 1.5 µL Lipofectamine 2000 (Invitrogen). Mouse Hepa1–6 cells or LA-4 cells were seeded on 24-well plates (3×10^4^ cells/wells), and then incubated at 37°C, in 5% CO_2_ for 24 hours. The culture medium was replaced by 400 µL of DMEM with 10%FBS without antibiotics, and then transfected with 98.5 µL siRNA or nkRNA or PnkRNA directed against mouse TGF-β1 in Opti-MEM (final concentration: 0.1, 0.3, 1, 3, 10 nM), using 1.5 µL Lipofectamine 2000. As negative controls, 100 µL Opti-MEM treatment or 1.5 µL Lipofectamine 2000 and 98.5 µL Opti-MEM treatment were used. The cells transfected with siRNA, nkRNA, and PnkRNA against mouse TGF-β1 were examined after 48 hours of transfection and used for RNA analysis. The experiments were performed in triplicates.

### RNA stability

To assess resistance to S7 nuclease, 60 picomoles of siRNA, nkRNA and PnkRNA directed against mouse TGF-β1 were incubated at 37°C in 50 µL of 50 mM Tris-HCl, pH 8, containing 5 mM CaCl_2_ and 0.5 units S7 nuclease (Roche Diagnostics, Japan). After the specified times, the S7 nuclease reactions were stopped and the samples were thawed and run on a 7 M Urea-15% polyacrylamide gel. The gel was then stained with SYBR Green II and analyzed with E-BOX-VX2 (M&S Instruments Inc., Japan) or ChemiDoc™ XRS+system (Biod-Rad Laboratories).

The stability of RNA therapeutic agents close to *in vivo* conditions was also evaluated. Each, siRNA, nkRNA or PnkRNA against mouse TGF-β1 (1 µg/5 µl) was added to 50 µl of mouse BALF drawn from mice and incubated at 37°C for 10 min. The RNA was then purified by phenol/chloroform and run on a 20% polyacrylamide gel. The gel was then stained using a solution of 1 µg/ml ethidium bromide (Sigma, St Louis, MO).and photographed using the UVP Bioimaging System (Upland, CA).

### Digestion by Dicer and analysis by MALDI-TOF mass spectrometry

nkRNA or PnkRNA directed against mouse TGF-β1, each 5 µg, was incubated with 5 units of ColdShock-Dicer (TaKaRa) in a reaction buffer (20 mM Tris-HCl, pH8.5, 150 mM NaCl, 2.5 mM MgCl_2_) at 37°C for 0, 1, 3 and 6 hours, and then 1 µg of 20 mM EDTA was added to stop the reaction. Each sample was then extracted with phenol/chloroform, and 100 ng of each was separated in 7 M urea denatured 15% PAGE and then stained using SYBR Green II RNA Gel Stain (Lonza). The samples were purified on ZipTipC18 reverse-phase microcolumns (Millipore), MALDI-TOF mas spectra were acquired in positive-ion mode with a matrix solution containing 10 mg/ml 3-hydroxypicolinic acid and 1 mg/ml diammonium citrate.

### Preparation of dumbbell-shaped RNA without nucleotide deletion

RNA without deleted nucleotide directed against human GAPDH was prepared as a single-stranded RNA, which then self-anneals into a structure containing 5′ and 3′ ends (**Figure 5**). The RNA 5′ end was phosphorylated by treating with T4 polynucleotide kinase (TaKaRa) in a reaction buffer containing 50 pmol RNA, enzyme 20 Units, 50 mM Tris-HCl, pH 8.0, 10 mM MgCl_2_, 5 mM DTT and 1 mM ATP at 37°C for 2 h. After phenol/chloroform extraction and ethanol precipitation, the RNA 3′ and 5′ ends were ligated and the RNA converted to a circular type by treating overnight with T4 RNA ligase (TaKaRa) at 16°C in a reaction buffer containing RNA 2 ug, Enzyme 50 U, 50 mM Tris-HCl, pH7.5, 10 mM MgCl_2_, 10 mM DTT, 1 mM ATP, 0.006% BSA. After phenol/chloroform extraction and ethanol precipitation, the formation of RNA was confirmed in 7 M urea-denatured 15% PAGE and by ESI-Q-TOF mass spectrometry. Dumbbell RNA was cut from the gel, extracted and purified using the small RNA Gel Extraction Kit (TaKaRa) following the manufacturer's instructions. The inhibitory activity of dumbbell RNA directed against human GAPDH was evaluated and compared with nkRNA in HCT 116 cell lines. The cells were treated with 1, 3, 10 nM of each RNA in the presence of lipofectamine 2000 and after 48 h mRNA was extracted from the cells and the amount of hGAPDH was quantified by real time PCR.

### Measurement of *Tm* values

*Tm* values of siRNA, nkRNA and PnkRNA in 50 mM phosphate buffer (PB), pH 7.5 or phosphate-buffered saline (PBS) were measured with UV-VIS spectrophotometers UV-1800 (Shimadzu Scientific Instruments., Japan), and analyzed with *Tm* Analysis software version 1,2,1,0 (Shimadzu Scientific Instruments, Japan).

### Quantitative real-time PCR

Total RNA was extracted from the cultured cells using RNeasy Mini Kit (Qiagen, Valencia, CA, USA), and cDNA was synthesized using SuperScript III (Invitrogen). To determine the mRNA knock-down level, Quantitative real-time PCR was carried out using the following primers (greiner Japan): human GAPDH, forward 5′-GGAGAAGGCTGGGGCTCATTTGC-3′ and reverse 5′-TGGCCAGGGGTGCTAAGCAGTTG-3′; human α-actin, forward 5′-GCCACGGCTGCTTCCAGCTCCTC-3′ and reverse 5′-AGGTCTTTGCGGATGTCCACGTCAC-3′, mouse TGF-β1, forward 5′-CCATTGCTGTCCCGTGCAGAGCTG-3′ and reverse 5′-ATGGTAGCCCTTGGGCTCGTGGATC-3′; mouse β-actin, forward 5′-GTCGTACCACAGGCATTGTGATGG-3′ and reverse 5′-GCAATGCCTGGGTACATGGTGG-3. Synthesized cDNAs were mixed with primers in Light Cycler Fast Start DNA Master SYBR Green I (Roche Diagnostics, Japan). Quantitative real-time PCR was performed using Light Cycler DX400 (Roche Diagnostics, Japan), and the reaction conditions were as follows: 95°C for 5 s, at 62°C for 15 s, and at 72°C for 15 s for 40 cycles (human GAPDH), or 95°C for 5 s, at 65°C for 15 s, and at 72°C for 15 s for 40 cycles (human β-actin, mouse TGF-β1, mouse β-actin). The data obtained from the assays were analyzed using Light Cycler Software version 4.1 (Roche Diagnostics, Japan). The mRNA knock-down levels were normalized with each β-actin transcript level.

### Animal model of acute lung injury (ALI)

Male C57/BL6 (8∼9 week-old, weight, 22∼24 g) mice purchased from Nihon SLC were used in the experiments. They were housed in the animal facility of Mie University, maintained on a constant 12-hour light/12-hour dark cycle in a temperature- and humidity-controlled room and were given food and water *ad libitum*. Under profound anesthesia (62.5 mg/kg intraperitoneal sodium pentobarbital) lung damage was induced by intratracheal (i.t.) instillation of LPS (5 mg/kg) dissolved in 50 µl of sterile saline (Nihon Kayaku) as previously described [24].

### Therapy of wild type mice with ALI using RNAi agents directed against TGF-β1

To assess the effect of nkRNA in acute lung injury, mice under anesthesia (intraperitoneal [i.p.] sodium pentobarbital) were treated with 100 µg of TGF-β1 nkRNA (nkRNA/LPS), scrambled nkRNA (control RNA/LPS), or vehicle (Vehicle/LPS) by intratracheal instillation one hour before LPS administration. Mice treated with i.p. injection of saline plus inhaled vehicle (vehicle/saline), TGF-β1 nkRNA (vehicle/TGF-β1 nkRNA) or scrambled nkRNA (vehicle/scrambled nkRNA) were used as control animals. The animals were sacrificed after 24 hours and BALF and lung tissue samples were taken for analysis.

For comparative analysis of the different RNAi agents, in a separate experiment, mice were treated with 100 µg of each siRNA (TGF-β1 siRNA/LPS), nkRNA (TGF-β1 nkRNA/LPS) and PnkRNA (TGF-β1 PnkRNA/LPS) directed against TGF-β1 or vehicle (vehicle/LPS) by i.t. instillation before LPS. Mice treated with i.p. injection of saline plus inhaled vehicle (vehicle/saline) were used as control animals. The animals were sacrificed after 24 hours and samples of BALF and lung tissue were taken for analysis.

### Therapy of human TGF-β1 TG mice with ALI using RNAi agents directed against TGF-β1

TGF-β1 bacterial artificial chromosome transgenic mice that express the human TGF-β1 gene specifically in the lungs under the control of the mouse SP-C promoter (hTGF-β1 TG) were used in these experiments. The hTGF-β1 TG mice spontaneously develop lung inflammation and fibrosis at 10 weeks of age as previously characterized [23]. To assess the therapeutic efficacy of RNAi agents in an ALI model closer to human conditions, hTGF-β1 TG mice were treated by i.t. instillation with 100 µg of each siRNA (hTGF-β1 siRNA/LPS), nkRNA (hTGF-β1 nkRNA/LPS) or PnkRNA (hTGF-β1 PnkRNA/LPS) directed against human TGF-β1 1 h before instillation of 100 µg LPS. Mice treated with vehicle (vehicle/LPS) and LPS were used as a positive control group and mice treated with 100 µg of control RNA (control RNA/LPS) and LPS were used as negative controls. Mice treated with i.p. injection of saline plus inhaled vehicle (vehicle/saline) were used as control animals. The animals were sacrificed after 24 h and BALF and lung tissue were taken for analysis.

### Therapy of wild type mice with pulmonary fibrosis using RNAi agents directed against TGF-β1

C57BL/6 wild type, female, 8 week-old (20∼22 g) mice were used in the experiments. They were purchased from Nihon SLC (Hamamatsu, Japan) and maintained in Mie University's animal house. The Committee for Animal Investigation of Mie University approved the experimental protocol. Lung fibrosis was induced by 100 mg/kg bleomycin (BLM) dissolved in sterile saline administered to randomized mice by constant subcutaneous infusion using osmotic minipumps (model 2001; Alzet Corporation, Palo Alto, CA) as previously described [23]; control mice received saline without BLM. RNAi agents against TGF-β1 (5 mg/kg) were administered i.t. on days 3, 7 and 14 after BLM. Controls were treated with i.t. scrambled RNAs (5 mg/kg) or vehicle. On the 21^st^ day after BLM administration, all animals were sacrificed by i.p. overdose of pentobarbital to collect BALF, blood and organs. The degree of lung fibrosis was assessed by Ashcroft score and the tissue content of collagen and hydroxyproline was measured as previously described [23], [25].

### BALF sampling

After euthanasia of the animals by i.p. anesthesia overdose, samples for biochemical and histological examinations were taken. Blood samples were collected by heart puncture and placed in tubes containing sodium citrate. Method for sampling of BALF in mice was previously described [23]. BALF total cell count was measured using a nucleocounter from ChemoMetec (Allerød, Denmark). The BALF was centrifuged and the supernatant was stored at −80°C until use for biochemical analysis. After cytospin separation of BALF cells, they were stained with May-Grunwald-Giemsa for differential cell counting (Merck, Darmstadt, Germany).

### Biochemical analysis

The level of protein was measured using commercial kits (BCA™ protein assay kit, Pierce, Rockford, IL) following the manufacturer's instructions. The levels of human and mouse TGF-β1 (R&D System, Minneapolis, MIN), interferon (IFN)-β and IFN-α (PBL Biomedical Laboratories, Piscataway, NJ) were measured using commercial EIA kits following the manufacturer's instructions.

### Lung histological findings

After euthanasia and thoracotomy, the pulmonary circulation was flushed with saline and then the lungs were excised. The lungs were perfused with 10% neutral buffered formalin, fixed in formalin and then embedded in paraffin. 5-µm thick sections of lung specimens were prepared and stained with Giemsa-eosin (Merck, Darmstadt, Germany) or hematoxylin-eosin and then examined under light microscopy (Olympus BX50 microscope, Tokyo, Japan).

### 5′-(Rapid Amplification of cDNA End) RACE assay

2 µg of total RNA from mouse lung tissue was used in the assays. First-strand cDNA synthesis was performed using the 5′-Full RACE Core Set (TAKARA) and the 5′ terminal-phosphorylated primer (5′-TGCAGGAGCGCACAA-3′). PCR was first performed using 1 µl of the first-strand cDNA synthesis reaction and primers (sense: 5′-AAGGTCCTTGCCCTCTACAACCAA-3′, antisense: 5′-TGTACTGTGTGTCCAGGCTCCAAA-3′). Reaction conditions were as follows: 98°C for 10 s, at 68°C for 30 s, and at 72°C for 30 s for 25 cycles. A nested PCR was then performed using 1 µl of the first-round reaction and primers (sense: 5′-TCCTTGCCCTCTACAACCAACACA-3′, antisense: 5′-TCCCAGACAGAAGTTGGCATGGTA-3′). Reaction conditions were as follows: 98°C for 10 s, at 68°C for 30 s, and at 72°C for 30 s for 35 cycles. Then, 10 µl of sample was analyzed on a 4% agarose gel.

## Supporting Information

Figure S1 **Representative HPLC chromatogram (black curves) and mass spectrum (red curves) of novel RNAi agents.** The purity and mass of each type of RNA are described in each corresponding box.
(PDF)

Figure S2 **Screening of mouse TGF-β1 nkRNA.** The inhibitory activity of varying concentrations of mouse TGF-β1 nkRNA and PnkRNA with deleted nucleotides (dn) at positions −2 (dn −2), −2 and −3 (dn −2, −3), 1 (dn 1) and 1 and 2 (dn 1, 2) was evaluated and compared; siRNA, nkRNA and PnkRNA caused a strong reduction in mTGF-β1 mRNA levels in Hepa 1–6 liver cells (A). nkRNA dn 1 and PnkRNA nd 1 against TGF-β1 also significantly decreased the target gene expression in lung epithelial cells (B). Statistical analysis by ANOVA. Data are expressed as the mean ± s.e.m. *p<0.05 vs cells treated with target nucleic acid agent.
(PDF)

Figure S3 **Digestion of nkRNA and PnkRNA by Dicer.** TGF-β1 nkRNA or PnkRNA, was incubated with Dicer as described under materials and methods for 0, 1, 3 and 6 h and degraded products were analyzed by mass spectrometry. Both nkRNA and PnkRNA were almost completely degraded by Dicer after 18 h (A). Mass spectrometry analysis (B, C) showed degradation products of 21∼22 mers (S1, S2, A1, A2, A3) in length, which are the candidate siRNAs of both nkRNA and PnkRNA as described in **Table S4**.
(PDF)

Figure S4 **Comparative efficacy of siRNA, nkRNA and PnkRNA against mouse TGF-β1 in acute lung injury and confirmation of target degradation.** The concentration of TGF-β1 was measured by enzyme immunoassay in lung tissue homogenates. All siRNA, nkRNA and PnkRNA reduced the expression of target mRNA expression in the model compared to untreated mice (A). 5′-RACE analysis confirmed target degradation in the lungs after treatment with each agent (B). Statistical analysis by ANOVA. Data are expressed as the mean ± s.e.m.
(PDF)

Table S1 **Sequence of human GAPDH nkRNA with deleted nucleotide (dn) at different positions on the sense strand.**
(DOC)

Table S2 **Sequence of mouse TGF-β1 nkRNA and PnkRNA with deleted nucleotides at different positions and sequence of mouse TGF-β1 siRNA.**
(DOC)

Table S3 **Sequence candidates and mass of fragments released after Dicer digestion.**
(DOC)

Table S4 **Sequence of siRNA, nkRNA and PnkRNA directed against mouse TGF-β1 mRNA.**
(DOC)

Table S5 **Sequence of siRNA, nkRNA and PnkRNA directed against human TGF-β1.**
(DOC)
