# Supplementary material for: Efficacy of a Novel Class of RNA Interference Therapeutic Agents
Source: PLoS One. 2012 Aug 15;7(8):e42655. doi: 10.1371/journal.pone.0042655 (PMC3419724; doi:10.1371/journal.pone.0042655)

Human GAPDH nkRNA dn 1

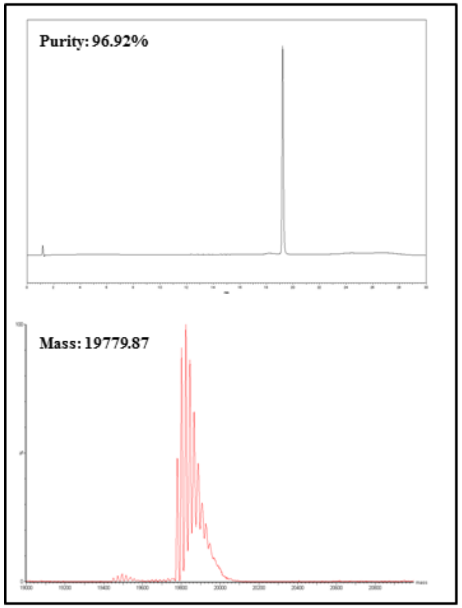

Human GAPDH PnkRNA nd 1

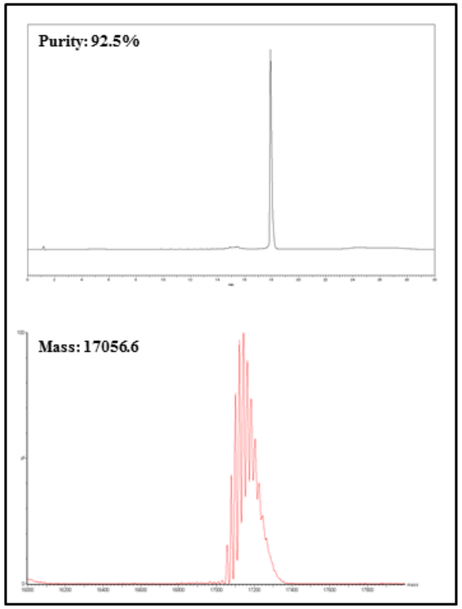

Human TGF-β1 nkRNA

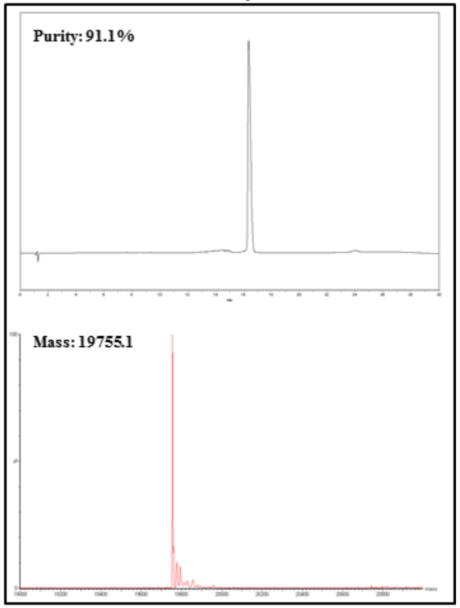

Human TGF-β1 PnkRNA

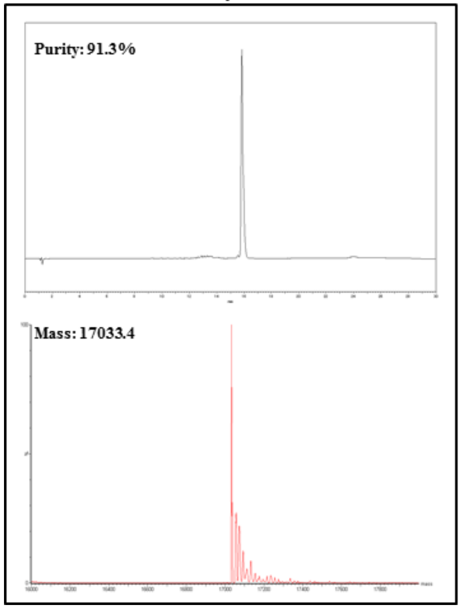

Mouse TGF-β1 nkRNA

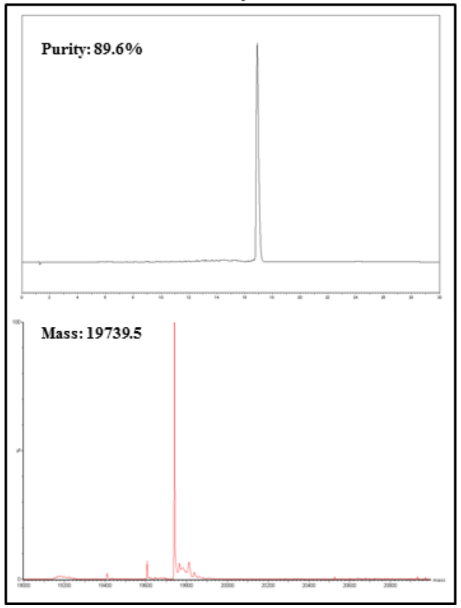

Mouse TGF-β1 PnkRNA

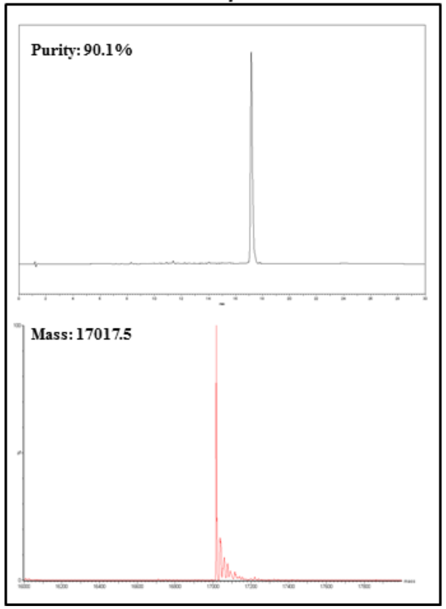

Supplement: Figure S1 — Representative HPLC chromatogram (black curves) and mass spectrum (red curves) of novel RNAi agents. The purity and mass of each type of RNA are described in each corresponding box. (PDF) [file pone.0042655.s001.pdf]
