# Supplementary figures and images for: Efficacy of a Novel Class of RNA Interference Therapeutic Agents
Source: PLoS One. 2012 Aug 15;7(8):e42655. doi: 10.1371/journal.pone.0042655 (PMC3419724; doi:10.1371/journal.pone.0042655)

**A**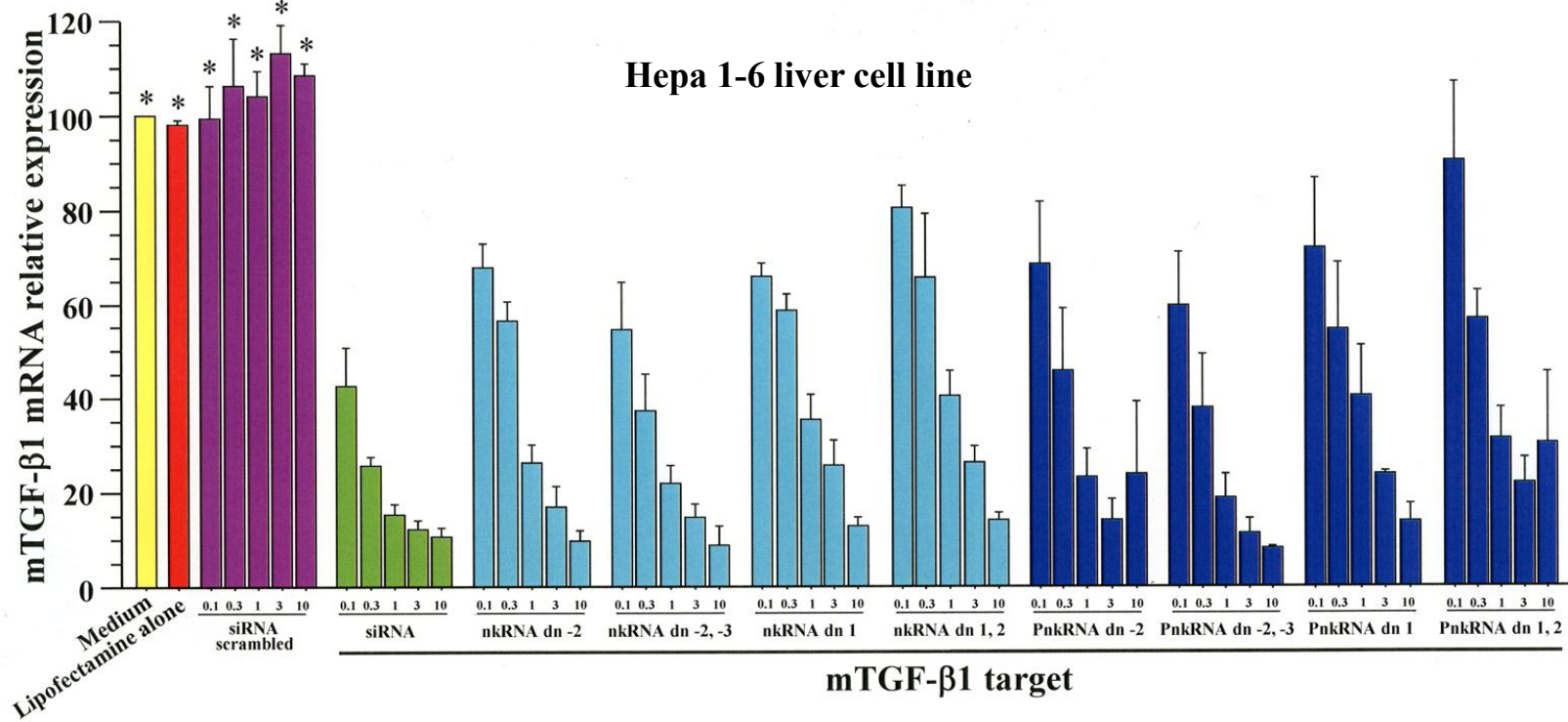**B**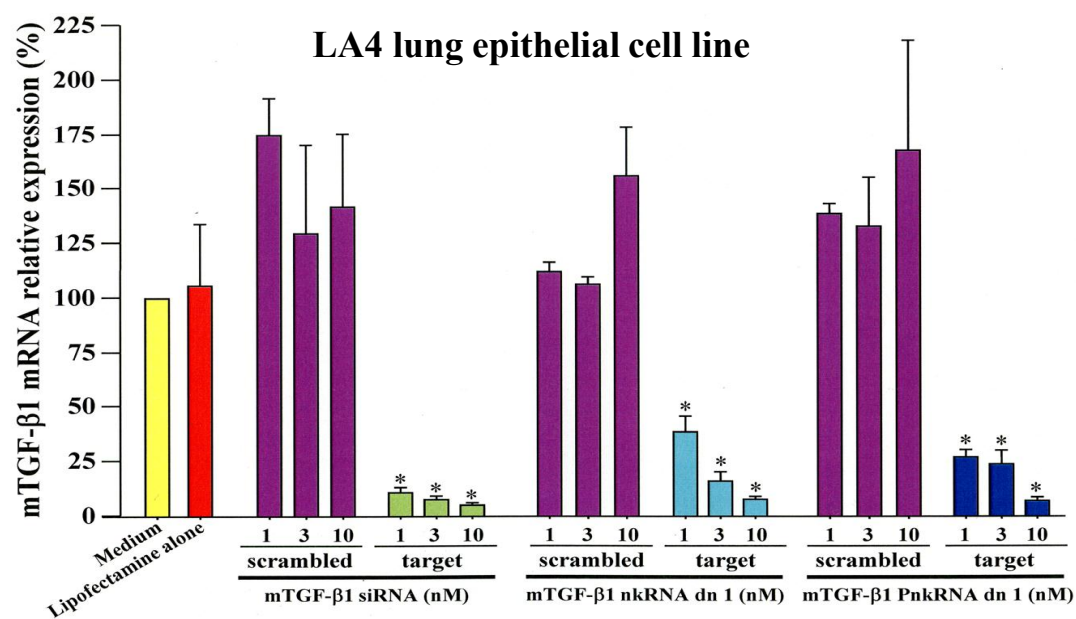

Supplement: Figure S2 — Screening of mouse TGF-β1 nkRNA. The inhibitory activity of varying concentrations of mouse TGF-β1 nkRNA and PnkRNA with deleted nucleotides (dn) at positions −2 (dn −2), −2 and −3 (dn −2, −3), 1 (dn 1) and 1 and 2 (dn 1, 2) was evaluated and compared; siRNA, nkRNA and PnkRNA caused a strong reduction in mTGF-β1 mRNA levels in Hepa 1–6 liver cells (A). nkRNA dn 1 and PnkRNA nd 1 against TGF-β1 also significantly decreased the target gene expression in lung epithelial cells (B). Statistical analysis by ANOVA. Data are expressed as the mean ± s.e.m. *p<0.05 vs cells treated with target nucleic acid agent. (PDF) [file pone.0042655.s002.pdf]

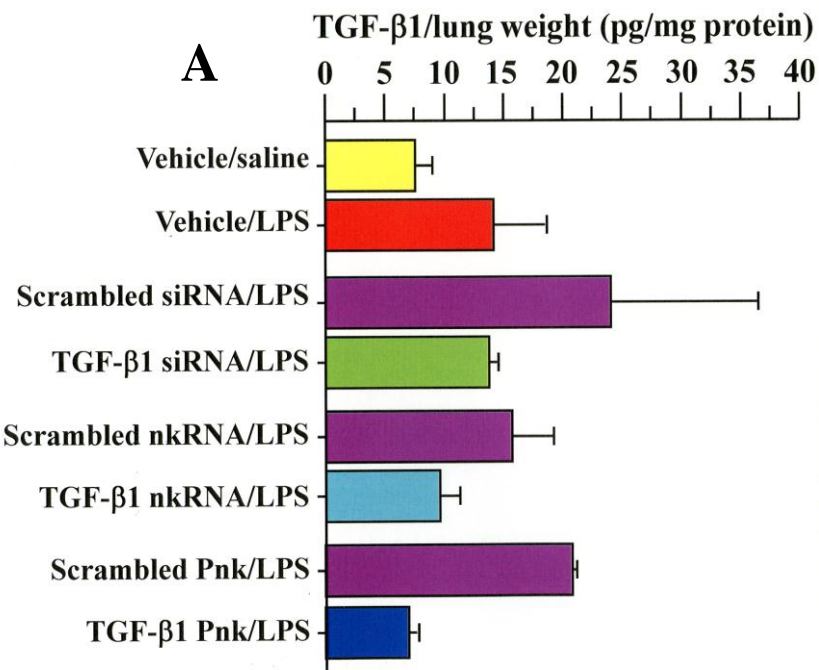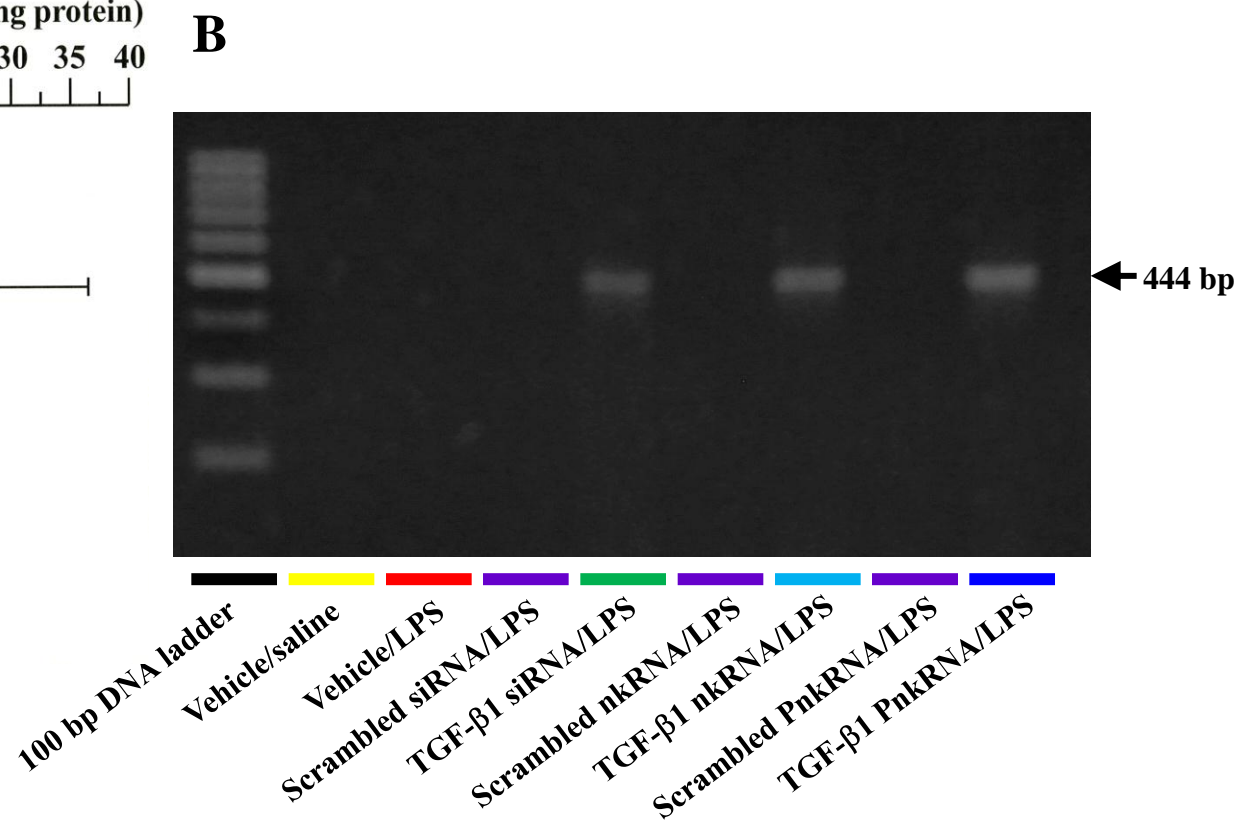

Supplement: Figure S4 — Comparative efficacy of siRNA, nkRNA and PnkRNA against mouse TGF-β1 in acute lung injury and confirmation of target degradation. The concentration of TGF-β1 was measured by enzyme immunoassay in lung tissue homogenates. All siRNA, nkRNA and PnkRNA reduced the expression of target mRNA expression in the model compared to untreated mice (A). 5′-RACE analysis confirmed target degradation in the lungs after treatment with each agent (B). Statistical analysis by ANOVA. Data are expressed as the mean ± s.e.m. (PDF) [file pone.0042655.s004.pdf]
