# Supplementary material for: Efficacy of a Novel Class of RNA Interference Therapeutic Agents
Source: PLoS One. 2012 Aug 15;7(8):e42655. doi: 10.1371/journal.pone.0042655 (PMC3419724; doi:10.1371/journal.pone.0042655)
Supplement: Figure S3 — Digestion of nkRNA and PnkRNA by Dicer. TGF-β1 nkRNA or PnkRNA, was incubated with Dicer as described under materials and methods for 0, 1, 3 and 6 h and degraded products were analyzed by mass spectrometry. Both nkRNA and PnkRNA were almost completely degraded by Dicer after 18 h (A). Mass spectrometry analysis (B, C) showed degradation products of 21∼22 mers (S1, S2, A1, A2, A3) in length, which are the candidate siRNAs of both nkRNA and PnkRNA as described in Table S4. (PDF) [file pone.0042655.s003.pdf]

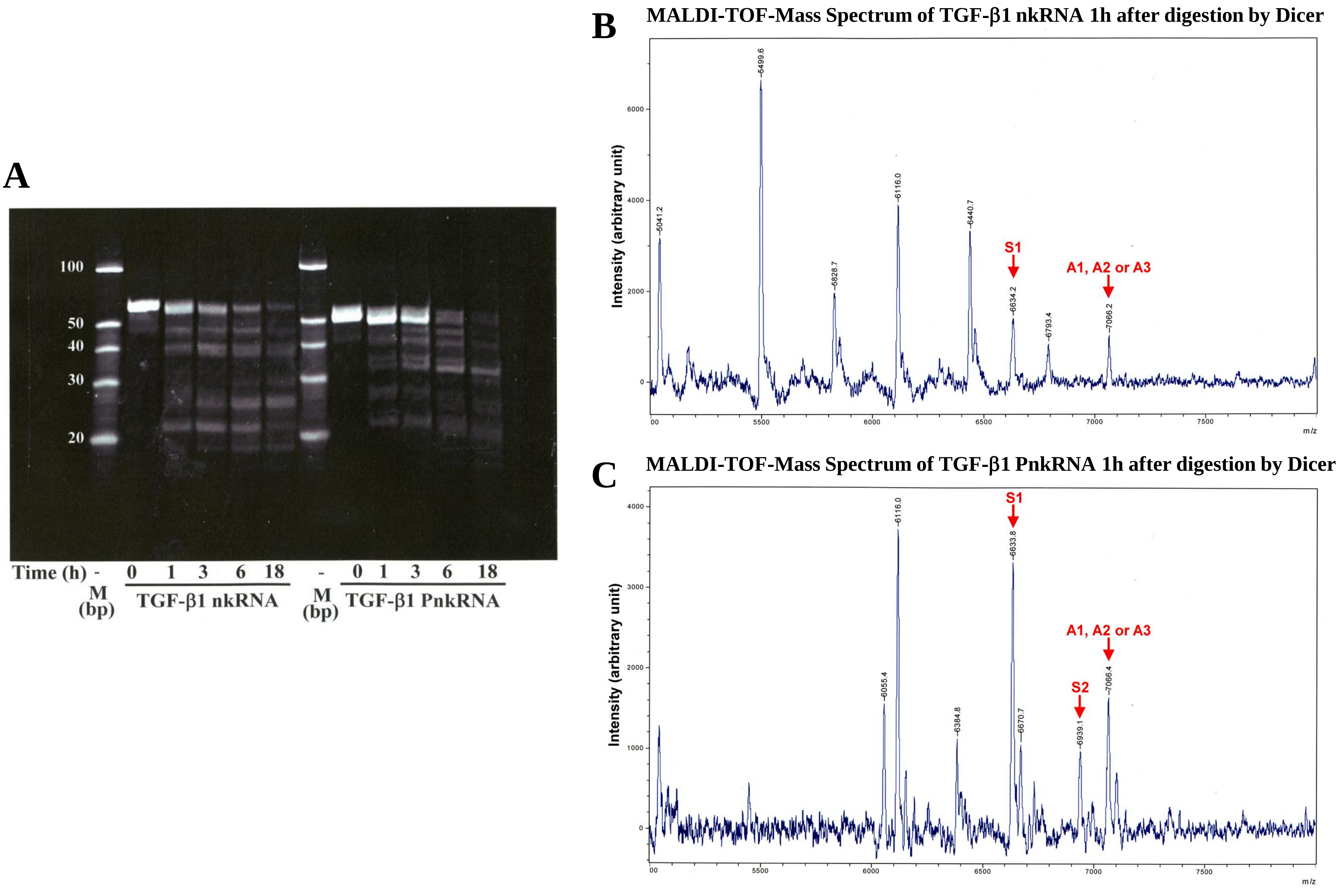
A
100
50
40
30
20
Time (h) - 0 1 3 6 18 - 0 1 3 6 18
M (bp)
TGF-β1 nkRNA
M (bp)
TGF-β1 PnkRNA
B
MALDI-TOF-Mass Spectrum of TGF-β1 nkRNA 1h after digestion by Dicer
Intensity (arbitrary unit)
5041.2
5499.6
5828.7
6116.0
6440.7
6634.2
6793.4
7066.2
S1
A1, A2 or A3
m/z
C
MALDI-TOF-Mass Spectrum of TGF-β1 PnkRNA 1h after digestion by Dicer
Intensity (arbitrary unit)
6055.4
6116.0
6384.8
6633.8
6670.7
6939.1
7066.4
S1
S2
A1, A2 or A3
m/z
