# Supplementary material for: Efficacy of a Novel Class of RNA Interference Therapeutic Agents
Source: PLoS One. 2012 Aug 15;7(8):e42655. doi: 10.1371/journal.pone.0042655 (PMC3419724; doi:10.1371/journal.pone.0042655)
Supplement: Table S1 — Sequence of human GAPDH nkRNA with deleted nucleotide (dn) at different positions on the sense strand. (DOC) [file pone.0042655.s005.doc]

| **Table S1. Sequence of human GAPDH nkRNA with deleted nucleotide (dn) at different positions on the sense strand** | | | |
| --- | --- | --- | --- |
| RNA class | Sequence | Mass | Purity (%) |
| dn -1 | ：5’- AACCAUGAGAAGUAUGACAACAGCCCCACACCGGCUGUUGUCAUACUUCUCAUGGUUCUUCG -3’ | 19739.5 | 96.6 |
| dn -2 | ：5’- ACCAUGAGAAGUAUGACAACAGCCCCACACCGGCUGUUGUCAUACUUCUCAUGGUUCUUCGG -3’ | 19755.7 | 96.3 |
| dn -3 | ：5’- CCAUGAGAAGUAUGACAACAGCCCCACACCGGCUGUUGUCAUACUUCUCAUGGUUCUUCGGA -3’ | 19755.7 | 92.3 |
| dn 1 | ：5’- CAUGAGAAGUAUGACAACAGCCCCACACCGGCUGUUGUCAUACUUCUCAUGGUUCUUCGGAA -3’ | 19779.9 | 96.9 |
| dn 2 | ：5’- AUGAGAAGUAUGACAACAGCCCCACACCGGCUGUUGUCAUACUUCUCAUGGUUCUUCGGAAC -3’ | 19779.8 | 93.5 |
| dn 3 | ：5’- UGAGAAGUAUGACAACAGCCCCACACCGGCUGUUGUCAUACUUCUCAUGGUUCUUCGGAACC -3’ | 19755.6 | 96.8 |
| dn 5 | ：5’- AGAAGUAUGACAACAGCCCCACACCGGCUGUUGUCAUACUUCUCAUGGUUCUUCGGAACCAU -3’ | 19739.8 | 95.4 |
| dn 7 | ：5’- AAGUAUGACAACAGCCCCACACCGGCUGUUGUCAUACUUCUCAUGGUUCUUCGGAACCAUGA -3’ | 19740.2 | 97.6 |
| dn 9 | ：5’- GUAUGACAACAGCCCCACACCGGCUGUUGUCAUACUUCUCAUGGUUCUUCGGAACCAUGAGA -3’ | 19755.9 | 96.5 |
| dn 11 | ：5’- AUGACAACAGCCCCACACCGGCUGUUGUCAUACUUCUCAUGGUUCUUCGGAACCAUGAGAAG -3’ | 19778.5 | 96.4 |
| dn 14 | ：5’- ACAACAGCCCCACACCGGCUGUUGUCAUACUUCUCAUGGUUCUUCGGAACCAUGAGAAGUAU -3’ | 19739.8 | 95.9 |
| dn 16 | ：5’- AACAGCCCCACACCGGCUGUUGUCAUACUUCUCAUGGUUCUUCGGAACCAUGAGAAGUAUGA -3’ | 19779.8 | 95.6 |
| dn 18 | ：5’- CAGCCCCACACCGGCUGUUGUCAUACUUCUCAUGGUUCUUCGGAACCAUGAGAAGUAUGACA -3’ | 19755.8 | 95.3 |
| dn 19 | ：5’- AGCCCCACACCGGCUGUUGUCAUACUUCUCAUGGUUCUUCGGAACCAUGAGAAGUAUGACAA -3’ | 19779.6 | 97.8 |
| dn 20 | ：5’- GCCCCACACCGGCUGUUGUCAUACUUCUCAUGGUUCUUCGGAACCAUGAGAAGUAUGACAAC -3’ | 19755.6 | 96.8 |
| dn 21 | ：5’- CCCCACACCGGCUGUUGUCAUACUUCUCAUGGUUCUUCGGAACCAUGAGAAGUAUGACAACA -3’ | 19739.8 | 96.5 |
| dn -4 | ：5’- CCCACACCGGCUGUUGUCAUACUUCUCAUGGUUCUUCGGAACCAUGAGAAGUAUGACAACAG -3’ | 19780.0 | 96.3 |
| dn -5 | ：5’- CCACACCGGCUGUUGUCAUACUUCUCAUGGUUCUUCGGAACCAUGAGAAGUAUGACAACAGC -3’ | 19779.8 | 93.3 |
| dn; deleted nucleotide | |  |  |
