# Supplementary material for: Efficacy of a Novel Class of RNA Interference Therapeutic Agents
Source: PLoS One. 2012 Aug 15;7(8):e42655. doi: 10.1371/journal.pone.0042655 (PMC3419724; doi:10.1371/journal.pone.0042655)
Supplement: Table S2 — Sequence of mouse TGF-β1 nkRNA and PnkRNA with deleted nucleotides at different positions and sequence of mouse TGF-β1 siRNA. (DOC) [file pone.0042655.s006.doc]

| **Table S2. Sequence of mouse TGF-b1 nkRNA and PnkRNA with deleted nucleotides at different positions and sequence of mouse TGF-b1 siRNA** | | | |
| --- | --- | --- | --- |
| RNA class | Sequence | Mass | Purity (%) |
| Target siRNA | ：5’- GCAGCUGUACAUUGACUUUAG -3’ (sense) / 5’-AAAGUCAAUGUACAGCUGCUU-3’ (antisense) | 6674.8 / 6681.9 | 94.9 / 98.1 |
| Scrambled siRNA | ：5’- GUGUCAGUGCUCAUUUACAAG -3’ (sense) / 5’-UGUAAAUGAGCACUGACACUU-3’ (antisense) | 6674.8 / 6681.9 | 98.3 / 97.9 |
| Target nkRNA dn -2 | ：5’- AGCAGCUGUACAUUGACUUUAGCCCCACACCGGCUAAAGUCAAUGUACAGCUGCUUCUUCGG-3’ | 19756.1 | 91.2 |
| Target nkRNA dn -2, -3 | ：5’- GCAGCUGUACAUUGACUUUAGCCCCACACCGGCUAAAGUCAAUGUACAGCUGCUUCUUCGG-3’ | 19426.6 | 93.5 |
| Target nkRNA dn 1 | ：5’- CAGCUGUACAUUGACUUUAGCCCCACACCGGCUAAAGUCAAUGUACAGCUGCUUCUUCGGAA-3’ | 19739.5 | 89.6 |
| Target nkRNA dn 1, 2 | ：5’- AGCUGUACAUUGACUUUAGCCCCACACCGGCUAAAGUCAAUGUACAGCUGCUUCUUCGGAA-3’ | 19434.7 | 90.3 |
| Target PnkRNA dn -2 | ：5’- AGCAGCUGUACAUUGACUUUAGCC-P-GGCUAAAGUCAAUGUACAGCUGCUUC-P-G-3’ | 17033.5 | 90.8 |
| Target PnkRNA -2, -3 | ：5’- GCAGCUGUACAUUGACUUUAGCC-P-GGCUAAAGUCAAUGUACAGCUGCUUC-P-G-3’ | 16704.3 | 92 |
| Target PnkRNA dn 1 | ：5’- CAGCUGUACAUUGACUUUAGCC-P-GGCUAAAGUCAAUGUACAGCUGCUUC-P-GAA-3’ | 17017.5 | 90.7 |
| Target PnkRNA dn 1, 2 | ：5’- AGCUGUACAUUGACUUUAGCC-P-GGCUAAAGUCAAUGUACAGCUGCUUC-P-GAA-3’ | 16712.3 | 91.9 |
