# Supplementary material for: Efficacy of a Novel Class of RNA Interference Therapeutic Agents
Source: PLoS One. 2012 Aug 15;7(8):e42655. doi: 10.1371/journal.pone.0042655 (PMC3419724; doi:10.1371/journal.pone.0042655)
Supplement: Table S3 — Sequence candidates and mass of fragments released after Dicer digestion. (DOC) [file pone.0042655.s007.doc]

| **Table S3. Sequence candidates and mass of fragments released after Dicer digestion** | | | | | | | |
| --- | --- | --- | --- | --- | --- | --- | --- |
| **Sense (S) Antis Antisense (A)** | **Sequence (5'→3')** | **Length** | **Calculated mass (a)** | **Observed mass (b)** | | | |
|  |  |  |  | **TGF-β1nkRNA** | **Δ: a - b** | **TGF-β1 PnkRNA** | **Δ: a - b** |
| S1 | CAGCUGUACAUUGACUUUAGC | 21 | 6634.0 | 6633.8 | (Δ= -0.2) | 6634.2 | (Δ= +0.2) |
| S2 | CAGCUGUACAUUGACUUUAGCC | 22 | 6939.2 | 6939.1 | (Δ= -0.1) | n.d. |  |
| A1 | pCUAAAGUCAAUGUACAGCUGCU | 22 | 7066.2 | 7066.4 | (Δ= +0.2) | 7066.2 | (Δ= 0) |
| A2 | pUAAAGUCAAUGUACAGCUGCUU | 22 | 7066.2 | 7066.4 | (Δ= -0.8) | 7066.2 | (Δ= -1.0) |
| A3 | pAAAGUCAAUGUACAGCUGCUUC | 22 | 7066.2 | 7066.4 | (Δ= +0.2) | 7066.2 | (Δ= 0) |

| **Structure of siRNAs based on the dicer-digestion products of nkRNA and PnkRNA** | | | |
| --- | --- | --- | --- |
| S1+A1 | CAGCUGUACAUUGACUUUAGC UCGUCGACAUGUAACUGAAAUC | S2+A1 | CAGCUGUACAUUGACUUUAGCC UCGUCGACAUGUAACUGAAAUC |
| S1+A2 | CAGCUGUACAUUGACUUUAGC UUCGUCGACAUGUAACUGAAAU | S2+A2 | CAGCUGUACAUUGACUUUAGCC UUCGUCGACAUGUAACUGAAAU |
| S1+A3 | CAGCUGUACAUUGACUUUAGC CUUCGUCGACAUGUAACUGAAA | S2+A3 | CAGCUGUACAUUGACUUUAGCC CUUCGUCGACAUGUAACUGAAA |
