# Supplementary material for: Efficacy of a Novel Class of RNA Interference Therapeutic Agents
Source: PLoS One. 2012 Aug 15;7(8):e42655. doi: 10.1371/journal.pone.0042655 (PMC3419724; doi:10.1371/journal.pone.0042655)
Supplement: Table S4 — Sequence of siRNA, nkRNA and PnkRNA directed against mouse TGF-β1 mRNA. (DOC) [file pone.0042655.s008.doc]

| **Table S4. Sequence of siRNA, nkRNA and PnkRNA directed against mouse TGF-β1 mRNA** | | | |
| --- | --- | --- | --- |
| RNA class | Sequence | Mass | Purity (%) |
| Target siRNA | ：5’- GCAGCUGUACAUUGACUUUAG -3’ (sense) / 5’-AAAGUCAAUGUACAGCUGCUU-3’ (antisense) | 6673.8 / 6680.8 | 94.7 / 99.8 |
| Scrambled siRNA | ：5’- GUGUCAGUGCUCAUUUACAAG -3’ (sense) / 5’-UGUAAAUGAGCACUGACACUU-3’ (antisense) | 6673.8 / 6680.8 | 98.1 / 97.6 |
| Target nkRNA dn 1 | ：5’- CAGCUGUACAUUGACUUUAGCCCCACACCGGCUAAAGUCAAUGUACAGCUGCUUCUUCGGAA-3’ | 19739.5 | 89.6 |
| Scrambled nkRNA | ：5’- UGUCAGUGCUCAUUUACAAGCCCCACACCGGCUUGUAAAUGAGCACUGACACUUCUUCGGAA-3’ | 19739.6 | 92.0 |
| Target PnkRNA dn 1 | ：5’- CAGCUGUACAUUGACUUUAGCC-P-GGCUAAAGUCAAUGUACAGCUGCUUC-P-GAA-3’ | 17017.5 | 90.7 |
| Scrambled PnkRNA | ：5’- UGUCAGUGCUCAUUUACAAGCC-P-GGCUUGUAAAUGAGCACUGACACUUC-P-GAA-3’ | 17017.5 | 92.2 |
