# Supplementary material for: Efficacy of a Novel Class of RNA Interference Therapeutic Agents
Source: PLoS One. 2012 Aug 15;7(8):e42655. doi: 10.1371/journal.pone.0042655 (PMC3419724; doi:10.1371/journal.pone.0042655)
Supplement: Table S5 — Sequence of siRNA, nkRNA and PnkRNA directed against human TGF-β1. (DOC) [file pone.0042655.s009.doc]

| **Table S5. Sequence of siRNA, nkRNA and PnkRNA directed against human TGF-β1** | | | |
| --- | --- | --- | --- |
| RNA class | Sequence | Mass | Purity (%) |
| Target siRNA | ：5’- GCAGAGUACACACAGCAUAUA-3’ （sense） / 5’- UAUGCUGUGUGUACUCUGCUU-3’ (antisense) | 6725.2 / 6603.8 | 98.3 / 90.7 |
| Control siRNA | ：5’-UACUAUUCGACACGCGAAGTT-3’ （sense） / 5’- CUUCGCGUGUCGAAUAGUATT-3’ (antisense) | 6652.8 / 6646.7 | 99.0 / 90.7 |
| Target nkRNA dn -2 | ：5’-AGCAGAGUACACACAGCAUAUACCCCACACCGGUAUAUGCUGUGUGUACUCUGCUUCUUCGG-3’ | 197755.1 | 91.1 |
| Control nkRNA | ：5’-AUACUAUUCGACACGCGAAGUUCCCCACACCGGAACUUCGCGUGUCGAAUAGUAUUCUUCGG-3’ | 19756.6 | 90.1 |
| Target PnkRNA dn -2 | ：5’-AGCAGAGUACACACAGCAUAUACC-P-GGUAUAUGCUGUGUGUACUCUGCUUC-P-G-3’ | 17033.4 | 91.3 |
| Control PnkRNA | ：5’-AUACUAUUCGACACGCGAAGUUCC-P-GGAACUUCGCGUGUCGAAUAGUAUUC-P-G-3’ | 17033.3 | 88.1 |
